# Global, regional, and national burdens of musculoskeletal disorders in postmenopausal women from 1990 to 2021 and projections to 2050: A population-based observational study using GBD 2021 data

**DOI:** 10.1097/MD.0000000000049611

**Published:** 2026-07-03

**Authors:** Zui Tian, Jingkai Di, Changjiang Mu, Zijian Guo, Yingda Qin, Zehui Yan, Chuan Xiang

**Affiliations:** a Department of Orthopedics, Second Hospital of Shanxi Medical University, Taiyuan, China.

**Keywords:** menopause, musculoskeletal disorders

## Abstract

Musculoskeletal disorders, the second-leading cause of disability from nonfatal diseases worldwide, pose significant economic burdens and public health challenges. Studies have demonstrated that the synergy between estrogen deficiency and inflammatory aging in postmenopausal women results in a higher risk of musculoskeletal disorders in women compared with men. This study aims to fill this gap by characterizing the burden of musculoskeletal disorders in postmenopausal women. Using data from the Global Burden of Disease Study 1990 to 2021, a comprehensive analysis was conducted on the incidence, prevalence, mortality, and disability-adjusted life years associated with musculoskeletal disorders in postmenopausal women. Additionally, levels of exposure to risk factors and their associated disease burdens were analyzed, along with projections to 2050. Globally, 476 million postmenopausal women were affected by musculoskeletal disorders in 2021, representing a 126.7% increase compared with 210 million in 1990. The projected number of prevalent cases is expected to reach 897 million by 2050. The burden of musculoskeletal disorders in menopausal women slowly increased with a higher Sociodemographic Index. A high body mass index was identified as the most significant risk factor for musculoskeletal disorders in menopausal women. The global burden of musculoskeletal disorders in menopausal women remains high, with substantial disparities between regions and socioeconomic strata. Prioritizing metabolic risk reduction, responding to demographic changes, and developing region-specific policies are essential for addressing this escalating public health challenge.

## 1. Background

Musculoskeletal disorders, the second-leading cause of disability from nonfatal diseases globally, have significantly increased worldwide in recent years, imposing substantial economic burdens and public health challenges on society.^[1,2]^ According to recent epidemiological data from the Global Burden of Disease Study (GBD), musculoskeletal disorders include rheumatoid arthritis, osteoarthritis, gout, neck pain, low back pain, and other related conditions.^[3,4]^ From 1990 to 2021, the global age-standardized prevalence of musculoskeletal disorders rose by 37.2%, resulting in an estimated loss of 149 million disability-adjusted life years (DALYs). Studies have demonstrated that estrogen deficiency in postmenopausal women synergizes with inflammatory aging, resulting in a 2.38-fold higher risk of musculoskeletal disorders in women compared with men.^[5,6]^ The number of postmenopausal women experiencing these disorders has dramatically increased worldwide, rising from 210 million in 1990 to 480 million in 2021. Consequently, musculoskeletal health has become increasingly threatened among women in midlife and older age. These diseases not only cause progressive pain syndromes, impaired motor function, and loss of occupational capability but also are significantly associated with comorbidities such as metabolic and psychological disorders. Patients with chronic musculoskeletal conditions have secondary anxiety and depression disorders.^[7,8]^ Despite the importance of these risk factors, research on musculoskeletal disorders in this population remains insufficient.

In this study, a comprehensive analysis was conducted on the incidence, prevalence, mortality, and DALYs associated with musculoskeletal disorders in postmenopausal women aged 55 years or older at the global, regional, and national levels, using data from the GBD from 1990 to 2021. Additionally, the relationship between SDI and the prevalence of these diseases was explored. Furthermore, a comprehensive analysis of risk factor exposure and associated disease burdens was performed, along with projections up to 2050. The study also addresses challenges and limitations related to the burden of musculoskeletal disorders in this population. The findings aim to increase public health awareness, provide insights into recent advancements, and offer a foundation for prevention and treatment strategies for musculoskeletal disorders in postmenopausal women.

## 2. Methods

### 2.1. Sources of information

All data for this study were obtained from the GBD 2021 database (https://vizhub.healthdata.org/gbd-results/). The database provides estimates for 371 diseases and 88 risk factors across 204 countries and territories and applies a standardized methodology for data collection and indicator assessment.^[9]^

Data on musculoskeletal disorders among postmenopausal women aged 55 years and older from 1990 to 2021 were extracted to assess temporal trends and disease burden. Extracted variables included demographic characteristics by age group (≥55 years), sex, year (1990–2021), geographic location (204 countries and territories, including member states of the World Health Organization, 5 Sociodemographic Index [SDI] regions, and 21 GBD regions), and SDI level. Based on the SDI, countries and territories were categorized into 5 development levels: low, low-middle, middle, high-middle, and high. The 21 GBD regions were defined according to epidemiologic similarity and geographic proximity.^[10]^

### 2.2. Analysis of indicators

The following metrics were used in this study to characterize the burden of musculoskeletal disorders in menopausal women aged 55 years and older: incidence, prevalence, and DALYs, along with their 95% uncertainty intervals (UIs). Age-standardized incidence, prevalence, and DALY rates were calculated based on the age structure of the GBD World Standard Population.

### 2.3. Statistical methods

Data on the burden of musculoskeletal disorders in menopausal women aged 55 years and older were analyzed globally for the period from 1990 to 2021 using Microsoft Excel (Microsoft Corporation) and software R (R Foundation for Statistical Computing). Data were cleaned, organized, statistically analyzed, and visualized using the dplyr and ggplot2 packages in R software (R Foundation for Statistical Computing). A *P*-value < .05 was considered statistically significant.

#### 2.3.1. Age group analysis

According to the age classification framework of GBD 2021, participants were categorized into 9 age groups: 55 to 59, 60 to 64, 65 to 69, 70 to 74, 75 to 79, 80 to 84, 85 to 89, 90 to 94, and 95 years or older. These categories were used to assess age-specific variations in the burden of musculoskeletal disorders among postmenopausal women.

#### 2.3.2. Trend analysis

Percentage change: Percentage changes in the burden of musculoskeletal disorders in menopausal women by age, sex, region, and country from 1990 to 2021 were calculated and visualized using R software. Trends in age-standardized rates were quantified by the estimated annual percentage change (EAPC). An EAPC and the lower limit of its 95% confidence interval (CI) >0 indicated an increasing trend, and vice versa.^[11]^

Joinpoint regression model: The Joinpoint regression model was used to analyze trends from 1990 to 2021 and to calculate the average annual percentage change (AAPC) and annual percentage change (APC) for each time period. APC trends were considered stable when the 95% CI included 0. An AAPC or APC significantly >0 or <0 indicated upward or downward trends, respectively.^[12]^ Model fitting and AAPC/APC calculations were performed using Joinpoint Regression Program (version 6.0.1; National Cancer Institute) and visualized using R.

#### 2.3.3. Correlation analysis

Pearson correlation analysis was used to assess the association between SDI and age-standardized rates of musculoskeletal disorders among postmenopausal women. In addition, the proportion of musculoskeletal disorders attributable to selected risk factors, including high body mass index, kidney dysfunction, and smoking, was evaluated.

### 2.4. Decomposition and projection analysis

#### 2.4.1. Decomposition analysis

Decomposition methods were applied to quantify the independent contributions of population growth, aging, and epidemiologic changes to the burden of musculoskeletal disorders among postmenopausal women while holding other factors constant.^[13]^

#### 2.4.2. Age-period-cohort analysis

The effects of age, period, and cohort on disease burden were evaluated using an age-period-cohort model. The age-period-cohort model was based on a Poisson distribution framework and decomposed age, period, and cohort effects to assess their associations with the risk of incidence and mortality from musculoskeletal disorders.^[14]^

To address the parameter estimation challenges arising from the linear dependency among age, period, and cohort effects in the age-period-cohort model, a Bayesian Markov chain Monte Carlo algorithm was incorporated. This model was then used to project the burden of musculoskeletal disorders among postmenopausal women from 2022 to 2050.^[15]^

### 2.5. Ethical statement

This study utilized publicly available, deidentified data from the GBD 2021 study. Consequently, no approval from an institutional review board or informed consent was required.

## 3. Results

### 3.1. Global burden of musculoskeletal disorders in postmenopausal women

In 2021, the age-standardized incidence of musculoskeletal disorders in postmenopausal women was 11,951.59 (95% UI: 9206.61, 15,124.70) per 100,000 population, the age-standardized prevalence was 60,482.07 (95% UI: 55,619.92, 65,532.64) per 100,000 population, and the age-standardized DALYs were 5722.74 (95% UI: 4007.39, 8045.78) per 100,000 person-years. Consequently, 94,043,144 (95% UI: 72,436,449, 119,062,247) new cases of musculoskeletal disorders were identified among postmenopausal women in 2021. The total number of affected postmenopausal women in 2021 was 476,105,959 (95% UI: 437,892,867, 515,791,046). DALYs attributable to musculoskeletal disorders in postmenopausal women in 2021 amounted to 45,039,176 (95% UI: 31,544,819, 63,312,778) person-years.

### 3.2. Regional burden of musculoskeletal disorders among postmenopausal women

In 2021, the high-SDI region had the highest age-standardized incidence rate (ASIR; 13,118.86, 95% UI: 10,322.75, 16,337.73), the highest age-standardized prevalence rate (ASPR; 65,506.71, 95% UI: 61,028.20, 69,984.57), and the highest age-standardized DALYs (7011.59, 95% UI: 4874.13, 9982.84) among the 5 SDI regions.

The middle-SDI region had the lowest ASIR (11,070.80, 95% UI: 8483.17, 14,075.48), while the low-SDI region had the lowest ASPR (54,980.29, 95% UI: 49,784.76, 60,627.54) and age-standardized DALYs (5212.94, 95% UI: 3673.72, 7296.48). Regarding temporal trends, the burden of musculoskeletal disorders in menopausal women increased across all SDI regions. Specifically, the ASIR in the high-SDI region (EAPC = 0.03, 95% UI: 0.02, 0.04), ASPR in the middle-SDI region (EAPC = 0.24, 95% UI: 0.21, 0.27), and age-standardized DALYs in the low-middle-SDI region (EAPC = 0.20, 95% UI: 0.16, 0.24) showed the most significant increases (Table 1).

**Table 1:** Age-standardized musculoskeletal disease burden outcomes for menopausal women in 5 SDI regions and 21 GBD regions.

Location	Incidence			Prevalence			DALYs		
	1990 (per 100,000 population, 95% UI)	2021 (per 100,000 population, 95% UI)	EAPCs (95% CI)	1990 (per 100,000 population, 95% UI)	2021 (per 100,000 population, 95% UI)	EAPCs (95% CI)	1990 (per 100,000 population, 95% UI)	2021 (per 100,000 population, 95% UI)	EAPCs (95% CI)
Global	12,561.68 (9570.39, 16,041.15)	11,951.59 (9206.61, 15,124.70)	−0.12 (−0.14, −0.10)	58,444.23 (53,332.73, 63,929.04)	60,482.07 (55,619.92, 65,532.64)	0.15 (0.13, 0.17)	5620.67 (3918.54, 7929.81)	5722.74 (4007.39, 8045.78)	0.11 (0.09, 0.13)
SDI									
High SDI	13,133.60 (10,083.33, 16,663.05)	13,118.86 (10,322.75, 16,337.73)	0.03 (0.01, 0.05)	62,499.17 (57,453.96, 67,804.75)	65,506.71 (61,028.20, 69,984.57)	0.17 (0.14, 0.19)	6146.17 (4307.42, 8676.47)	6404.97 (4536.61, 8954.95)	0.15 (0.13, 0.17)
High-middle SDI	13,326.55 (10,149.82, 16,981.07)	12,274.80 (9395.03, 15,601.12)	−0.21 (−0.24, −0.19)	58,549.17 (53,115.06, 64,277.10)	59,667.94 (54,585.98, 64,884.72)	0.13 (0.10, 0.15)	5613.72 (3879.93, 7971.82)	5554.25 (3831.91, 7870.46)	0.04 (0.01, 0.07)
Middle SDI	11,636.64 (8823.13, 14,887.11)	11,070.80 (8483.17, 14,075.48)	−0.08 (−0.12, −0.03)	55,836.02 (50,728.17, 61,266.59)	58,690.71 (53,735.84, 63,752.62)	0.24 (0.21, 0.27)	5223.43 (3607.89, 7382.56)	5374.50 (3731.39, 7583.32)	0.19 (0.16, 0.22)
Low-middle SDI	11,950.16 (9032.42, 15,321.86)	11,651.63 (8829.48, 14,902.49)	−0.09 (−0.14, −0.05)	56,030.06 (50,750.73, 61,779.50)	59,339.72 (54,226.99, 64,833.09)	0.19 (0.16, 0.21)	5455.97 (3858.04, 7583.08)	5793.18 (4111.14, 8035.69)	0.20 (0.16, 0.24)
Low SDI	11,948.24 (9033.40, 15,354.98)	11,582.24 (8772.05, 14,866.21)	−0.11 (−0.13, −0.09)	53,103.71 (47,801.27, 58,986.37)	54,980.29 (49,784.76, 60,627.54)	0.11 (0.10, 0.13)	5057.44 (3560.92, 7087.83)	5212.94 (3673.72, 7296.48)	0.11 (0.08, 0.15)
21 GBD regions									
Andean Latin America	9224.10 (7068.62, 11,674.25)	9489.04 (7298.09, 11,982.85)	0.12 (0.10, 0.13)	55,689.26 (50,890.42, 60,540.77)	59,168.69 (54,527.28, 63,944.21)	0.21 (0.20, 0.22)	4884.84 (3372.02, 6971.45)	5247.73 (3657.36, 7427.74)	0.26 (0.24, 0.27)
Australasia	14,555.24 (11,088.15, 18,604.66)	14,786.59 (11,225.18, 18,844.86)	0.06 (0.03, 0.09)	65,273.02 (59,969.19, 70,757.59)	67,870.57 (62,577.18, 73,421.68)	0.13 (0.11, 0.14)	6747.87 (4792.66, 9388.10)	6989.63 (4904.37, 9850.07)	0.10 (0.06, 0.14)
Caribbean	9534.42 (7279.35, 12,154.10)	9657.90 (7458.86, 12,159.05)	0.06 (0.05, 0.07)	51,965.27 (47,397.00, 56,572.31)	54,703.82 (50,107.40, 59,179.09)	0.18 (0.18, 0.19)	4478.16 (3080.85, 6394.22)	4684.39 (3245.57, 6680.95)	0.17 (0.16, 0.18)
Central Asia	12,739.23 (9606.11, 16,360.39)	12,847.58 (9705.29, 16,507.75)	0.02 (0.01, 0.03)	57,176.91 (51,420.21, 63,147.95)	61,525.93 (55,404.76, 67,520.90)	0.26 (0.23, 0.28)	5259.80 (3583.13, 7558.89)	5607.05 (3836.00, 8070.54)	0.24 (0.22, 0.27)
Central Europe	14,508.34 (10,972.51, 18,544.71)	14,535.11 (11,005.42, 18,638.42)	0.00 (−0.01, 0.00)	59,459.50 (53,387.73, 66,059.81)	61,791.18 (55,967.68, 68,123.34)	0.14 (0.13, 0.14)	5901.72 (4079.93, 8350.15)	6074.37 (4192.06, 8639.10)	0.11 (0.10, 0.12)
Central Latin America	10,644.23 (8071.82, 13,620.99)	10,580.08 (8052.88, 13,491.33)	0.01 (−0.03, 0.04)	58,564.64 (53,505.80, 63,916.31)	62,049.82 (57,150.28, 67,201.24)	0.19 (0.19, 0.20)	5686.54 (4016.78, 7917.81)	6024.52 (4256.20, 8385.76)	0.20 (0.18, 0.22)
Central Sub-Saharan Africa	11,408.20 (8598.43, 14,751.49)	11,241.08 (8482.46, 14,488.84)	−0.06 (−0.07, −0.05)	52,469.38 (47,142.57, 58,125.98)	53,191.68 (47,934.91, 58,792.75)	0.02 (0.00, 0.05)	4777.14 (3287.96, 6801.92)	4829.32 (3347.03, 6888.86)	0.02 (0.01, 0.04)
East Asia	11,832.74 (8963.08, 15,127.95)	10,714.13 (8298.06, 13,521.24)	−0.14 (−0.21, −0.06)	55,194.53 (49,960.41, 60,818.93)	56,962.62 (52,090.63, 62,012.10)	0.27 (0.21, 0.33)	5103.33 (3497.95, 7288.42)	5020.84 (3458.72, 7155.53)	0.15 (0.08, 0.23)
Eastern Europe	15,289.32 (11,570.34, 19,555.22)	15,315.82 (11,646.03, 19,545.00)	0.02 (0.01, 0.02)	62,601.04 (56,445.72, 69,075.38)	64,283.86 (58,436.33, 70,489.80)	0.15 (0.12, 0.17)	6130.70 (4195.52, 8761.65)	6347.36 (4379.84, 8999.23)	0.19 (0.15, 0.23)
Eastern Sub-Saharan Africa	12,186.11 (9208.80, 15,690.45)	11,972.90 (9062.23, 15,396.15)	−0.06 (−0.07, −0.04)	51,696.20 (46,249.91, 57,804.97)	53,436.34 (48,151.46, 59,169.74)	0.11 (0.10, 0.12)	4805.75 (3323.67, 6813.48)	4893.24 (3359.24, 6948.53)	0.08 (0.07, 0.09)
High-income Asia Pacific	13,880.76 (10,658.05, 17,671.94)	13,412.30 (10,324.16, 16,995.35)	−0.08 (−0.11, −0.05)	67,588.22 (62,530.36, 72,784.24)	70,214.88 (65,236.22, 75,177.08)	0.24 (0.14, 0.33)	6835.03 (4770.69, 9714.31)	7011.59 (4874.13, 9982.84)	0.18 (0.10, 0.26)
High-income North America	12,740.47 (9836.69, 16,116.09)	12,860.92 (10,427.31, 15,514.71)	0.06 (0.04, 0.08)	65,141.17 (60,217.77, 70,259.44)	68,746.86 (64,816.12, 72,665.16)	0.11 (0.08, 0.15)	6452.50 (4553.81, 9059.53)	6795.94 (4896.34, 9372.25)	0.11 (0.09, 0.13)
North Africa and Middle East	11,786.67 (8953.69, 15,113.82)	12,021.71 (9164.47, 15,414.07)	0.07 (0.07, 0.08)	53,975.49 (48,606.80, 59,779.17)	58,565.92 (53,314.22, 64,162.91)	0.27 (0.26, 0.28)	5190.61 (3570.13, 7277.54)	5608.26 (3862.55, 7851.55)	0.27 (0.26, 0.28)
Oceania	10,853.44 (8200.76, 14,006.99)	10,967.51 (8322.25, 14,057.96)	0.05 (0.03, 0.07)	51,258.90 (46,236.25, 56,647.47)	53,491.74 (48,476.45, 58,721.80)	0.14 (0.14, 0.15)	4570.38 (3128.24, 6490.93)	4666.89 (3187.21, 6715.27)	0.09 (0.08, 0.10)
South Asia	12,453.88 (9385.30, 16,028.65)	11,799.96 (8920.85, 15,120.50)	−0.20 (−0.29, −0.12)	58,475.30 (53,134.93, 64,367.68)	62,162.18 (56,865.05, 67,803.41)	0.20 (0.16, 0.24)	5765.64 (4122.04, 7986.72)	6114.42 (4360.36, 8454.81)	0.19 (0.11, 0.26)
Southeast Asia	10,377.25 (7853.17, 13,332.59)	10,423.85 (7920.71, 13,364.26)	0.03 (0.02, 0.04)	49,132.25 (44,176.63, 54,524.49)	52,929.39 (47,988.50, 58,195.05)	0.24 (0.24, 0.25)	4541.33 (3140.03, 6351.71)	4848.95 (3348.65, 6834.56)	0.22 (0.22, 0.23)
Southern Latin America	13,123.21 (9941.28, 16,810.31)	13,563.74 (10,268.14, 17,408.05)	0.09 (0.05, 0.13)	66,284.09 (61,034.63, 71,868.77)	69,098.29 (63,871.15, 74,445.73)	0.10 (0.07, 0.14)	6860.77 (4839.07, 9689.38)	7243.77 (5124.83, 10,058.58)	0.13 (0.07, 0.19)
Southern Sub-Saharan Africa	11,802.38 (8972.40, 15,120.62)	11,322.96 (8620.71, 14,509.72)	−0.09 (−0.11, −0.08)	55,040.17 (49,866.71, 60,447.14)	56,733.61 (51,663.69, 61,981.99)	0.13 (0.12, 0.14)	5146.36 (3569.75, 7297.15)	5162.53 (3599.31, 7281.20)	0.06 (0.04, 0.08)
Tropical Latin America	11,931.55 (9070.52, 15,293.49)	12,597.78 (9630.99, 16,020.26)	0.16 (0.13, 0.19)	60,752.82 (55,450.69, 66,276.86)	63,462.07 (58,266.75, 68,793.73)	0.11 (0.09, 0.13)	5855.48 (4096.10, 8220.45)	6142.05 (4292.79, 8609.91)	0.10 (0.06, 0.14)
Western Europe	13,058.79 (10,031.17, 16,554.24)	13,437.94 (10,227.77, 17,176.60)	0.12 (0.10, 0.14)	58,845.52 (53,798.50, 64,225.95)	60,849.39 (55,712.32, 66,215.41)	0.10 (0.10, 0.11)	5650.86 (3925.48, 7975.96)	5812.29 (4009.11, 8289.53)	0.09 (0.07, 0.10)
Western Sub-Saharan Africa	11,339.02 (8596.80, 14,512.55)	11,120.39 (8467.08, 14,227.96)	−0.07 (−0.09, −0.04)	51,899.68 (46,757.29, 57,372.36)	53,848.16 (48,780.82, 59,085.35)	0.13 (0.12, 0.13)	4734.15 (3268.27, 6709.71)	4875.53 (3355.15, 6894.51)	0.11 (0.10, 0.12)

CI = confidence interval, DALYs = disability-adjusted life years, EAPC = estimated annual percentage change, GBD = Global Burden of Disease Study, SDI = Sociodemographic Index, UI = uncertainty interval.

Among the 21 GBD regions in 2021, Eastern Europe had the highest ASIR (15,315.82, 95% UI: 11,646.03, 19,545.00), high-income Asia Pacific had the highest ASPR (70,214.88, 95% UI: 65,236.22, 75,177.08), and Southern Latin America had the highest age-standardized DALYs (7243.77, 95% UI: 5124.83, 10,058.58). Conversely, Andean Latin America had the lowest ASIR (9489.04, 95% UI: 7298.09, 11,982.85), Southeast Asia had the lowest ASPR (49,132.25, 95% UI: 44,176.63, 54,524.49), and Oceania had the lowest age-standardized DALYs (4666.89, 95% UI: 3187.21, 6715.27). Temporal trends revealed that the disease burden increased across all GBD regions. Among these, the ASIR in Tropical Latin America (EAPC = 0.16, 95% UI: 0.13, 0.19), ASPR in East Asia (EAPC = 0.27, 95% UI: 0.21, 0.33), and age-standardized DALYs in North Africa and the Middle East (EAPC = 0.27, 95% UI: 0.26, 0.28) showed the most significant increases (Table 1).

### 3.3. National and regional burden of musculoskeletal disorders among postmenopausal women

**Figure 1.**
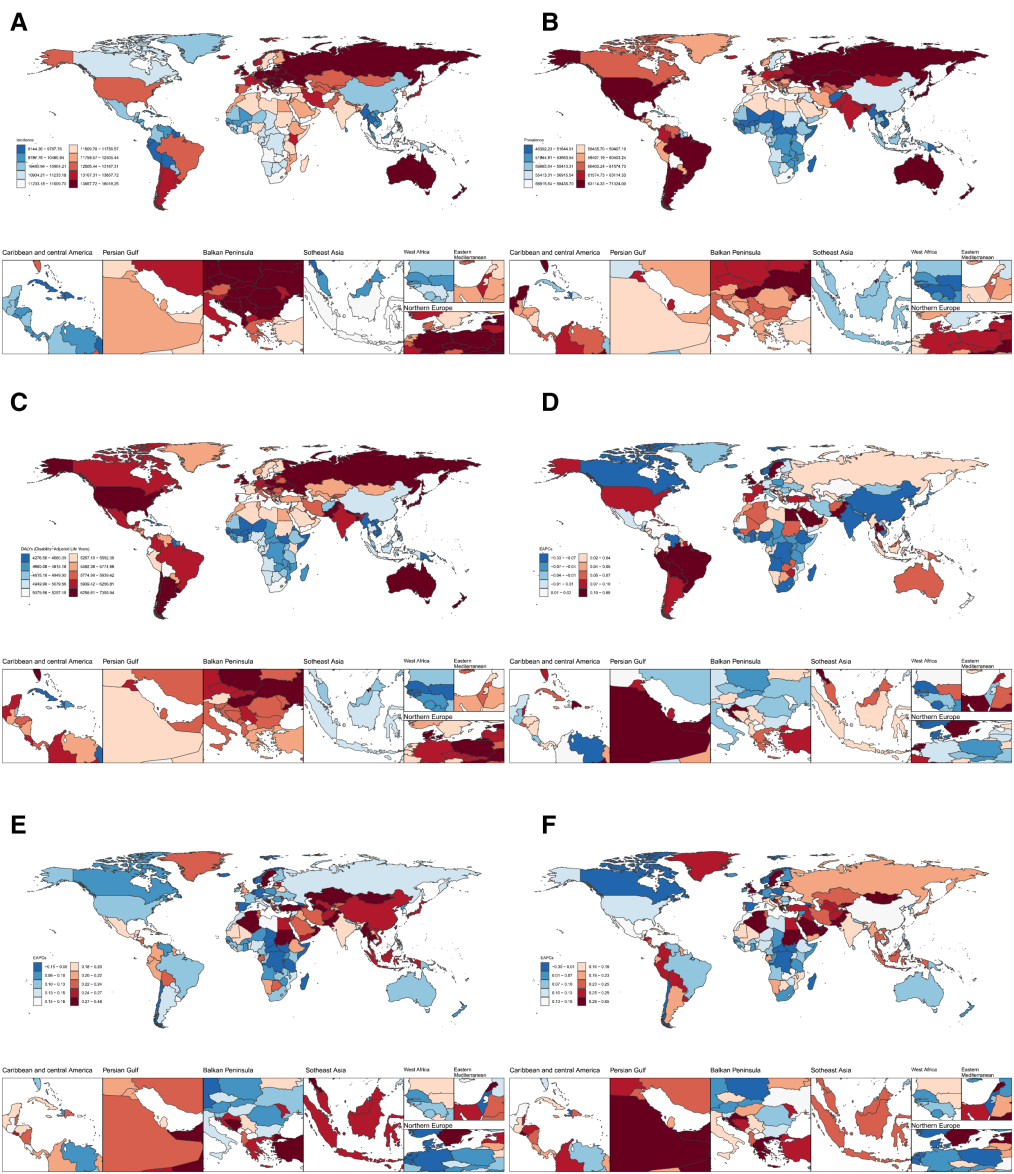
The burden of musculoskeletal diseases among menopausal women in 204 countries and regions around the world in 2021. (A) ASIR; (B) ASPR; (C) age-standardized DALYs; (D) EAPC of ASIR; (E) EAPC of ASPR; (F) EAPC of age-standardized DALYs. ASIR = age-standardized incidence rate, ASPR = age-standardized prevalence rate, DALYs = disability-adjusted life years, EAPC = estimated annual percentage change.

In 2021, Ukraine had the highest ASIR of musculoskeletal disorders in menopausal women (15,859.65, 95% UI: 11,962.35, 20,318.83 per 100,000 population). Japan had the highest ASPR (70,617.82, 95% UI: 65,704.12, 75,602.46 per 100,000 population), and Chile had the highest age-standardized DALYs (7283.11, 95% UI: 5155.30, 10,105.58 per 100,000 person-years). In contrast, Myanmar had the lowest ASIR (9236.72, 95% UI: 6964.54, 11,879.17 per 100,000 population), Eritrea had the lowest ASPR (48,881.04, 95% UI: 43,288.35, 54,576.13 per 100,000 population), and Haiti had the lowest age-standardized DALYs (4319.76, 95% UI: 3005.93, 6118.78 per 100,000 person-years; Fig. 1, Supplementary Table 1, Supplemental Digital Content 1). Longitudinal trend analysis showed decreasing trends in the burden of musculoskeletal disorders among menopausal women in Madagascar, Israel, Burundi, and the Democratic Republic of the Congo. Additionally, ASIRs decreased in 53 countries, ASPRs in 5 countries, and age-standardized DALYs in 13 countries. However, the burden increased in the remaining countries and regions (Fig. 1, Supplementary Table 1, Supplemental Digital Content 1). Notably, the ASIR in the United Kingdom (EAPC = 0.67, 95% UI: 0.57, 0.77), ASPR in Equatorial Guinea (EAPC = 0.48, 95% UI: 0.45, 0.51), and age-standardized DALYs in Taiwan (EAPC = 0.64, 95% UI: 0.57, 0.71) showed the most pronounced increases. Detailed disease burden data for 204 countries and regions are presented in Supplementary Table 1, Supplemental Digital Content 1.

### 3.4. Age-sex-time association analysis of musculoskeletal disorders among postmenopausal women

**Figure 2.**
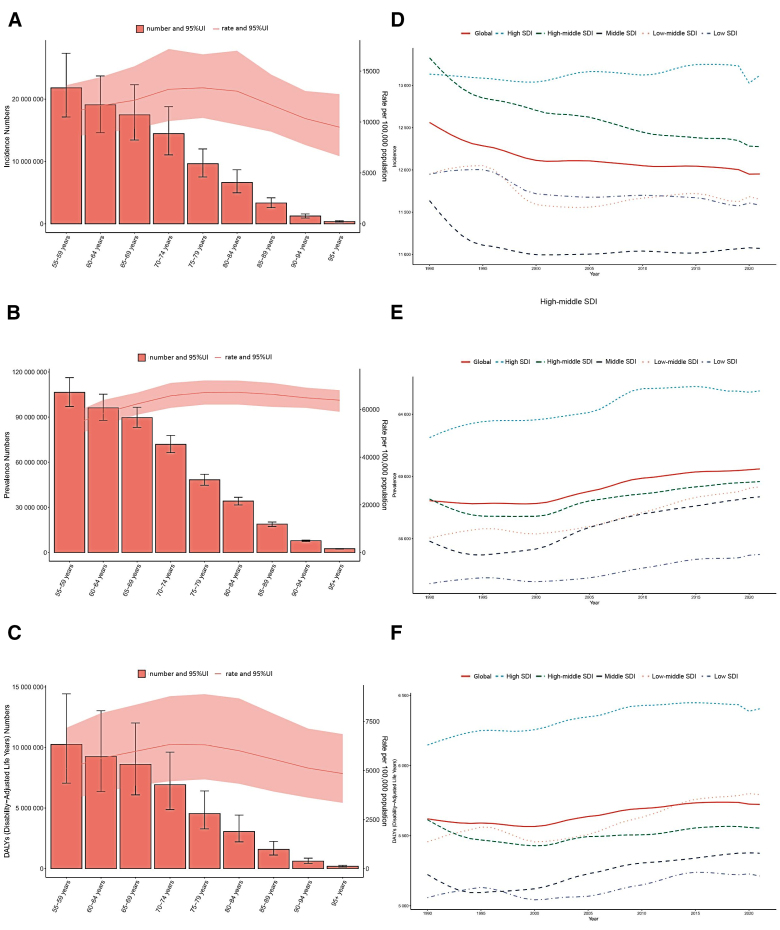
Age-sex trend of the burden of musculoskeletal diseases in menopausal women and results of Joinpoint regression analysis. (A) Incidence; (B) prevalence; (C) DALYs; (D) Joinpoint analysis of incidence; (E) Joinpoint analysis of prevalence; (F) Joinpoint analysis of DALYs. DALYs = disability-adjusted life years, SDI = Sociodemographic Index.

In 2021, the prevalence of musculoskeletal disorders increased with age among postmenopausal women aged 55 to 84 years and decreased in those aged 85 years and older (Fig. 2). The incidence increased with age in the 55 to 74-year group, remained stable in the 75 to 84-year group, and decreased in the 85+ group. Age-standardized DALY rates increased with age in the 55 to 74-year group and decreased in those aged 75 years and older.

Analysis of temporal trends from 1990 to 2021 indicated heterogeneous patterns across age groups. Globally, the ASIR decreased across all age groups. The age-standardized ASPR increased in all age groups over the same period. Age-standardized DALY rates decreased in women aged 55 to 79 years but increased among those aged 80 years and older (Figs. 4–6).

**Figure 3.**
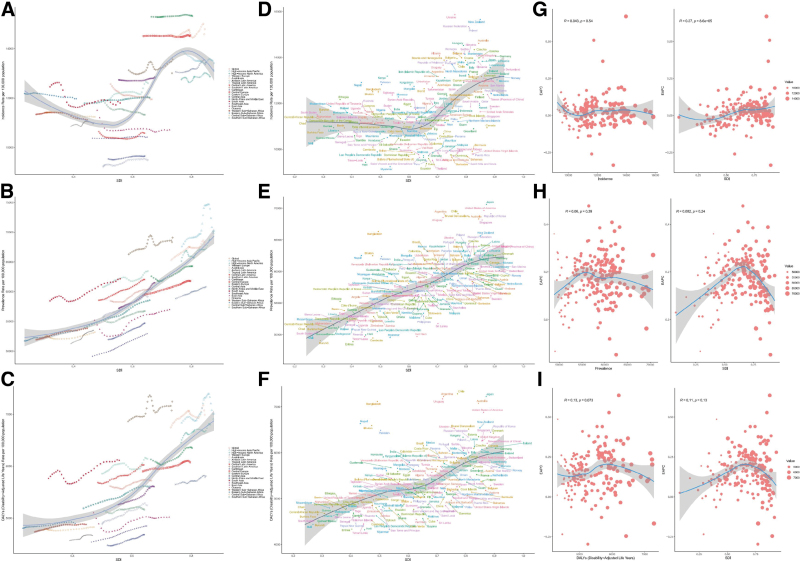
SDI analysis results. (A) Incidence of 21 regions; (B) prevalence of 21 regions; (C) DALYs of 21 regions; (D) incidence of 204 countries; (E) prevalence of 204 countries; (F) DALYs of 204 countries; (G) EAPC of incidence; (H) EAPC of prevalence; (I) EAPC of DALYs. DALYs = disability-adjusted life years, EAPC = estimated annual percentage change, SDI = Sociodemographic Index.

### 3.5. Trends in the burden of musculoskeletal disorders in menopausal women

Joinpoint regression analysis indicated that, between 1990 and 2021, the incidence, prevalence, and DALYs of musculoskeletal disorders in menopausal women generally increased. Specifically, the AAPC for incidence was −18.836 (95% CI: −19.74, −17.94), with significant changes occurring around 1993, 2000, and 2018. The AAPC for prevalence was 67.013 (95% CI: 65.17, 68.86), with significant changes concentrated in 2000, 2009, and 2015. The AAPC for DALYs was 3.526 (95% CI: 3.29, 3.76), with notable changes around 2000, 2008, and 2016 (Fig. 2). Since 2015, the increase in disease burden has slightly slowed but remains significantly elevated.

### 3.6. Association between SDI and musculoskeletal disorders among postmenopausal women

Across the 21 GBD regions and globally, the SDI was positively associated with the ASIR, ASPR, and age-standardized DALY rates of musculoskeletal disorders among postmenopausal women.

Among the 204 countries included in the analysis, incidence (*r* = 0.4566, *P* = 7.54 × 10^−12^), prevalence (*r* = 0.7084, *P* < .001), and DALY rates (*r* = 0.7067, *P* < .001) all increased with increasing SDI. Overall, the disease burden demonstrated a gradual upward trend with higher SDI levels. At comparable SDI levels, countries in Southeast Asia had consistently lower disease burdens than those in other regions (Fig. 3).

Further analysis of temporal trends showed that the EAPC of incidence was positively and linearly associated with SDI. In contrast, the associations between SDI and the EAPC of prevalence and DALY rates were nonlinear, both peaking at an SDI of approximately 0.7 and subsequently declining with further increases in SDI (Fig. 3).

### 3.7. Age-period-cohort analysis of the burden of musculoskeletal disorders among postmenopausal women

**Figure 4.**
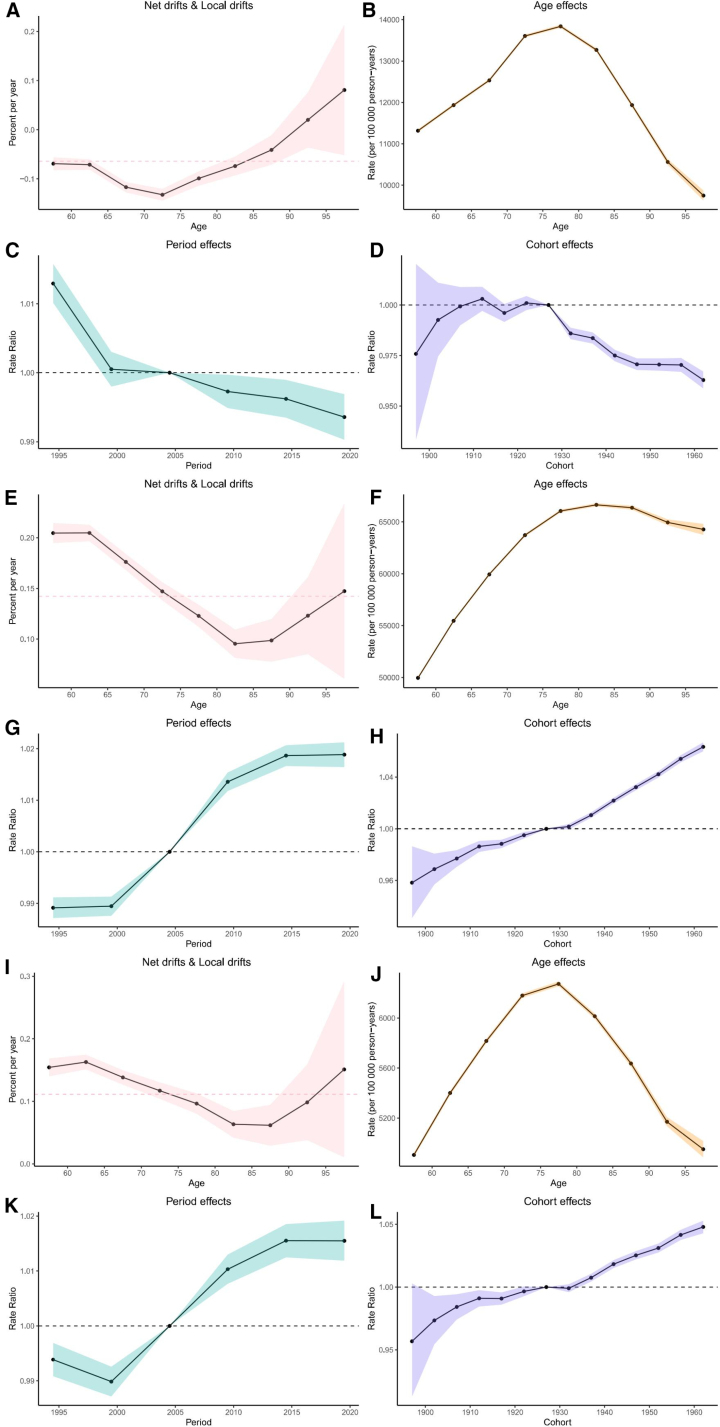
Results of age-period cohort analyses. (A) Net drifts and local drifts for incidence; (B) age effect for incidence; (C) period effect for incidence; (D) cohort effect for incidence; (E) net drifts & local drifts for prevalence; (F) age effect for prevalence; (G) period effect for prevalence; (H) cohort effect for prevalence; (I) net drifts & local drifts for DALYs; (J) age effect for DALYs; (K) period effect for DALYs; (L) cohort effect for DALYs. DALYs = disability-adjusted life years.

Age-period-cohort analysis of incidence, prevalence, and age-standardized DALY rates showed similar patterns. The age effect indicated that disease burden increased with age, peaking at approximately 80 years, and subsequently declined (Fig. 4).

Period effects on incidence and prevalence demonstrated a progressive increase in disease burden from 1990 to 2021 (Fig. 4). Cohort effects indicated that later-born cohorts experienced higher incidence and prevalence rates than earlier-born cohorts (Fig. 4).

### 3.8. Decomposition analysis of the burden of musculoskeletal disorders among postmenopausal women

Decomposition analysis indicated that, globally, across the 5 SDI regions and the 21 GBD regions, population growth, aging, and epidemiologic changes contributed to the burden of musculoskeletal disorders in postmenopausal women in similar patterns.

**Figure 5.**
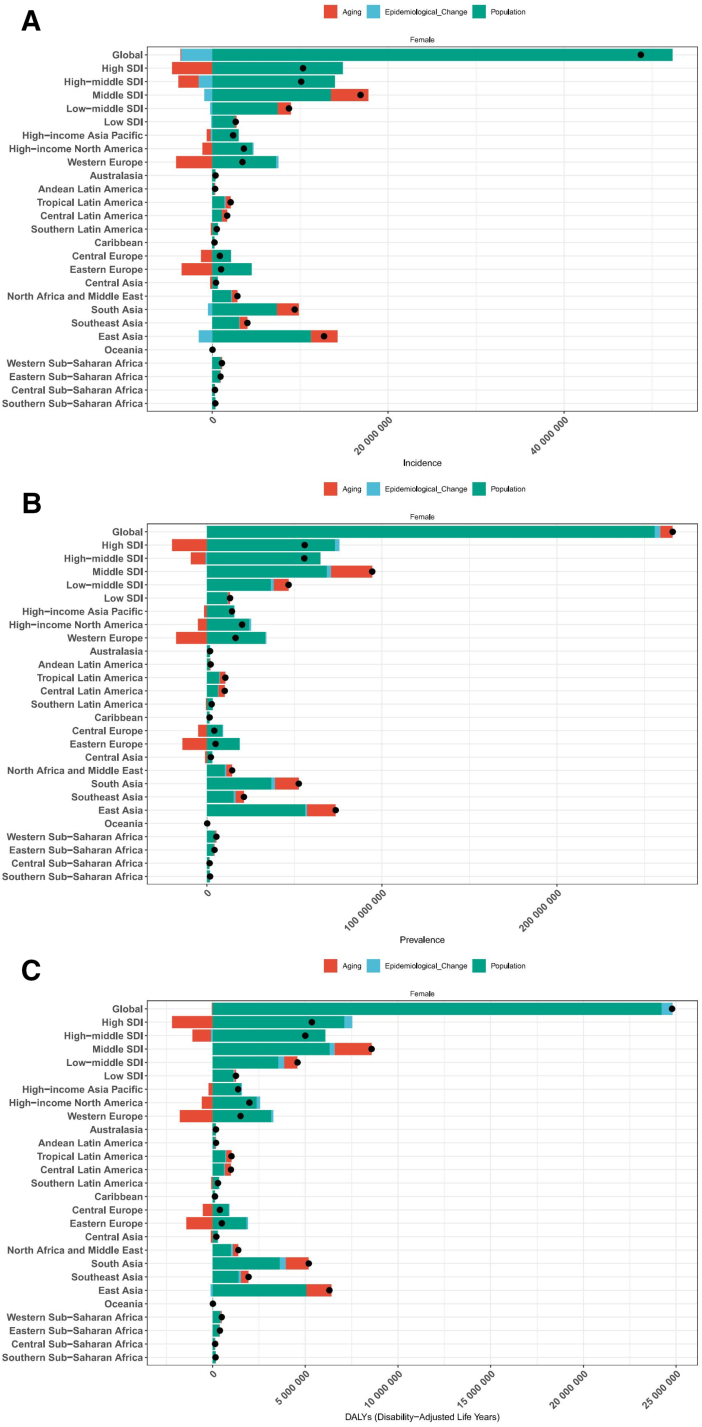
Results of decomposition analysis. (A) Incidence for global, 5 SDI and 21 GBD regions; (B) prevalence for global, 5 SDI and 21 GBD regions; (C) DALYs for global, 5 SDI and 21 GBD regions. DALYs = disability-adjusted life years, GBD = Global Burden of Disease Study, SDI = Sociodemographic Index.

Specifically, in most regions, population growth, aging, and epidemiologic changes all contributed to increases in disease burden, with population growth being the largest contributor (Fig. 5). Exceptions were observed in high-SDI and high-middle-SDI regions, as well as in high-income Asia Pacific, high-income North America, Western Europe, Central Europe, Eastern Europe, and Central Asia, where aging contributed to a reduction in disease burden.

### 3.9. Predictive analysis of musculoskeletal disorder burden in menopausal women

**Figure 6.**
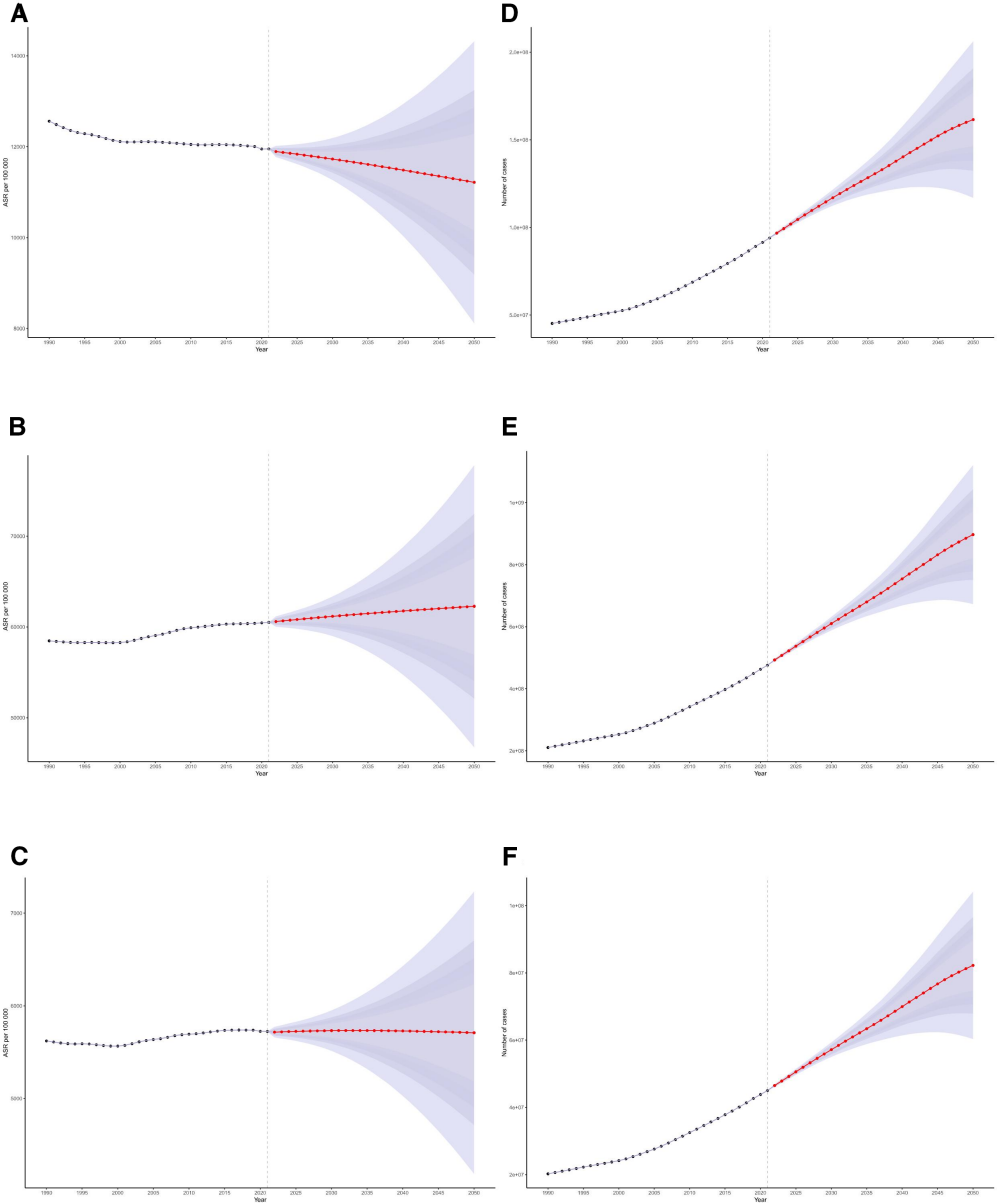
Results of predictive analysis. (A) ASIR; (B) ASPR; (C) age-standardized DALYs; (D) the actual value of incidence; (E) the actual value of prevalence; (F) the actual value of DALYs. ASIR = age-standardized incidence rate, ASPR = age-standardized prevalence rate, DALYs = disability-adjusted life years.

Projection analysis suggested that the global burden of musculoskeletal disorders in menopausal women will continue to rise from 2022 to 2050. By 2050, the ASIR, ASPR, and age-standardized DALYs for musculoskeletal disorders in menopausal women will reach 11,216.65 per 100,000 population, 62,273.91 per 100,000 population, and 5709.63 per 100,000 person-years, respectively. Consequently, 161,554,286 new cases of musculoskeletal disorders are expected among menopausal women aged 55 years and older by 2050, with 896,936,194 cases in total, resulting in a loss of 102,552 life-years (Fig. 6).

### 3.10. Risk factors for DALYs

**Figure 7.**
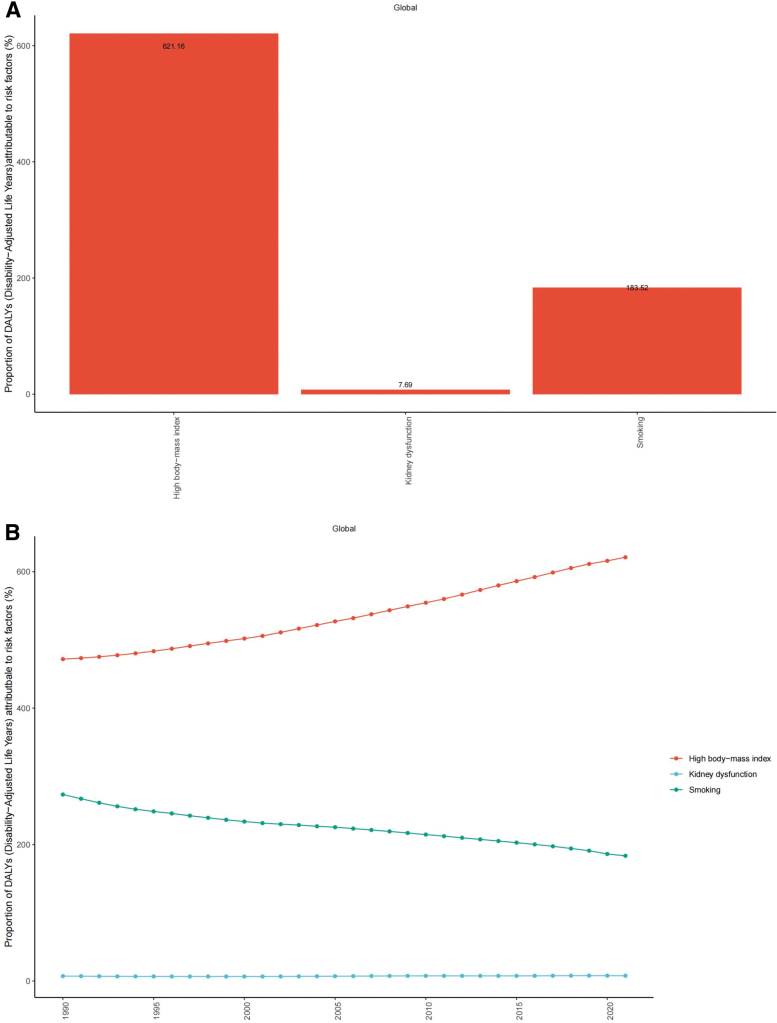
Risk factors for DALYs of musculoskeletal disorders in menopausal women worldwide and in 5 SDI regions (A) Risk factors for musculoskeletal disorders in menopausal women in 2021; (B) the time trend of risk factors for musculoskeletal disorders in menopausal women. DALYs = disability-adjusted life years, SDI = Sociodemographic Index.

Only risk factors for DALYs associated with musculoskeletal disorders among postmenopausal women were assessed in this analysis. These included high body mass index, kidney dysfunction, and smoking. High body mass index was the leading risk factor globally and across the 5 SDI regions and 21 GBD regions (Fig. 7).

Over time, the DALY burden attributable to metabolic risks, particularly high body mass index, showed an increasing trend, whereas the burden attributable to smoking demonstrated a declining trend (Fig. 7). Detailed results are summarized in the corresponding tables.

## 4. Discussion

Musculoskeletal disorders, the second-leading cause of disability from nonfatal diseases globally,^[9]^ have markedly increased in recent years, posing substantial economic and public health challenges. Evidence indicates that the synergy between estrogen deficiency and inflammatory aging places postmenopausal women at a higher risk compared with men.^[16,17]^ Considering population aging and projected disease prevalence, the number of postmenopausal women affected is expected to rise from 476 million in 2021 to 896 million in 2050. Thus, this study focuses on postmenopausal women to characterize epidemiological patterns and underlying mechanisms of musculoskeletal disorders, aiming to guide evidence-based management of bone health in this high-risk population.

Time-series analysis demonstrated that the burden of metabolic-related musculoskeletal disorders increased in all regions over the past 3 decades, with metabolic risk consistently identified as the primary contributor, accounting for approximately 50% of attributable risk among postmenopausal women. The synergistic interaction between hormonal fluctuations and metabolic abnormalities plays a critical role in this elevated risk. Estrogen, a key regulator of bone resorption and formation, declines significantly after menopause, disrupting bone metabolic homeostasis, accelerating bone resorption, impairing bone formation, and rapidly reducing bone mineral density.^[17,18]^ Concurrently, lower estrogen levels impair muscle protein synthesis, leading to muscle atrophy and reduced strength.^[5,19]^ Reduced estrogen diminishes osteoprotection by 50%, while oxidative stress in a hyperglycemic environment further accelerates bone microstructural degradation.^[20,21]^ This combined effect results in a higher risk of osteoporotic fractures among women aged 55 years and older compared with men of the same age. Given these findings, regular medical checkups are recommended for all postmenopausal women aged over 55 to identify metabolic risks early. A joint metabolic-bone health screening system should be established, incorporating blood pressure monitoring, glycosylated hemoglobin testing, and lipid profiling into routine examinations. Additionally, timely treatment of individuals with metabolic abnormalities through pharmacological interventions (e.g., ACE inhibitors and SGLT2 inhibitors) combined with resistance exercise is advised to comprehensively mitigate the progression from metabolic risks to musculoskeletal complications.

Three decades of global surveillance data indicate that high BMI increasingly contributes to musculoskeletal disorders in postmenopausal women. Multiple mechanisms are involved in this impact. First, obesity-induced mechanical overload accelerates joint degeneration, leading to rapid wear of articular cartilage and a higher risk of osteoarthritis.^[22]^ Second, adipose tissue disrupts cartilage and bone metabolism by secreting inflammatory factors, such as leptin, adiponectin, tumor necrosis factor-alpha, and interleukin-6.^[18,23]^ Additionally, excessive obesity increases intramuscular fat deposition, causing decreased muscle strength and mass.^[24]^ A study reported that moderate obesity may benefit bone health, with hip and leg fat potentially protective for bone density.^[25]^ This dose-dependent effect indicates that clinical interventions must balance weight management and bone health. Given the rising global burden of musculoskeletal disorders, healthcare measures and public education should be strengthened globally and regionally. Personalized health management programs based on BMI stratification, combined exercise and nutritional interventions, and public education campaigns to optimize weight perception are recommended for better patient management.

Significant regional differences exist in musculoskeletal disorders among postmenopausal women. This study demonstrated a linear relationship between disease burden and the SDI. The ASIR, ASPR, and age-standardized DALYs gradually increased with higher SDI. Observational data from 204 countries revealed that developed countries, such as the United Kingdom, had higher increases in ASIRs (EAPC of ASIR). This suggests that disease management for musculoskeletal disorders in postmenopausal women is closely related to national economic status. Such differences may result from the widespread availability of imaging diagnostic tools in high-SDI regions, improving detection of conditions like early degenerative joint lesions, osteoporosis, and subclinical disorders (e.g., sarcopenia and fibromyalgia). Conversely, the shortage of healthcare professionals and medical equipment in low-income areas likely leads to missed or misdiagnosed cases, exacerbating disease burden disparities.^[10]^ Additionally, the growing elderly population in high-SDI regions^[26]^ creates a dual burden of increased longevity and chronic disease. Notably, an accelerated life pace in these regions may elevate chronic stress levels, with prolonged cortisol secretion reducing osteoprotegerin expression.^[27]^ Furthermore, occupational competition significantly elevates anxiety disorder prevalence in women aged 50 and older compared with 30 years ago,^[28]^ and psychological stress accelerates bone loss through the hypothalamic-pituitary-adrenal axis.^[29]^ Therefore, regional socioeconomic development levels should be fully considered when developing disease prevention strategies. Efforts should include strengthening diagnostic knowledge and targeted health interventions for regions with insufficient medical resources.

Decomposition analysis revealed that population growth, aging, and epidemiologic changes consistently influenced the burden of musculoskeletal disorders in postmenopausal women, with population growth being the most significant contributor. Over the past 30 years, aging has notably reduced disease burden in high-income regions, including high SDI, high-middle SDI, high-income Asia Pacific, high-income North America, Western Europe, Central Europe, and Eastern Europe. Conversely, in other regions, population growth, aging, and epidemiologic changes increased disease burden. This trend may reflect the synergistic effects of better healthcare resource integration, systematic health interventions, and social policies in high-income regions, which emphasize elderly health management.^[30]^ Additionally, statistically, the true disease burden of aging populations may be partially obscured by the healthy survivor effect (advanced medical care extending healthy life expectancy) and standardization of disease coding. Collectively, these multidimensional factors generate a unique scenario described as “decoupling aging from disease burden.”

This paper investigated the relative effects of age, period, and birth cohort on trends in the incidence, prevalence, and DALYs of musculoskeletal disorders in menopausal women. Age effect analysis indicated that the burden of musculoskeletal disorders significantly increased with age, peaking around age 80. Mortality also rose with age. Aging, as one of the most prominent risk factors for musculoskeletal disorders, significantly affects women’s health. Age-related degenerative changes in the musculoskeletal system directly weaken mobility and balance,^[31]^ increasing the risk of falls and fractures.^[32]^ Gynecological disorders (e.g., endometritis, uterine fibroids, and ovarian tumors) in postmenopausal women can trigger low back pain,^[33]^ indirectly affecting the musculoskeletal system. Additionally, chronic low-grade inflammation and metabolic abnormalities (e.g., insulin resistance and vitamin D deficiency) caused by aging further exacerbate musculoskeletal degeneration through the release of inflammatory factors^[34]^ and interference with glucose and calcium-phosphorus metabolism, forming a vicious cycle. Under these combined effects, the risk of musculoskeletal disorders in postmenopausal women continues to rise. Therefore, attention should be paid to aging-related health challenges, and targeted preventive measures should be implemented.

Due to the time-cohort effect, the global incidence of musculoskeletal disorders among postmenopausal women aged 55 years and older has decreased. This phenomenon may primarily result from environmental changes and shifts in work patterns. With accelerating global industrialization, work has gradually shifted from intensive physical labor to electronic and information-based tasks, reducing long-term physical stress on the musculoskeletal system. Regarding cohort effects, individuals in later-born cohorts had lower incidence risks than those in earlier-born cohorts. Two main reasons explain this difference: first, earlier-born cohorts may have accumulated more disease-related risk factors, such as prolonged physical labor and limited nutritional conditions. Second, health conditions during early-life stages have long-term effects on musculoskeletal disorders, and later-born cohorts likely benefited from improved early-life healthcare, resulting in reduced disease risk.

Although the ASIRs of musculoskeletal disorders in menopausal women decreased across age groups, cohort analysis showed increases in ASPRs and DALYs. This paradoxical phenomenon may reflect the dual nature of current disease control strategies. On one hand, preventive measures successfully reduced new cases; on the other hand, disease burden among existing cases continued to rise due to population aging, prolonged chronic disease duration, and changes in quality of life. Despite declining ASIR, increasing ASPR and age-standardized DALYs highlight the need to strengthen prevention, early diagnosis, and comprehensive treatment strategies for musculoskeletal disorders.

Particularly, the increasing prevalence indicates a significant deterioration of the musculoskeletal disorder epidemic, emphasizing the urgent need for enhanced disease control. Distinguishing between period and cohort trends helps identify core drivers behind prevalence changes. Specifically, net drift, a composite indicator reflecting calendar time and continuous birth cohort trends, provides essential insights into underlying mechanisms. Overall, the global APC analysis of musculoskeletal disorders reveals the complex dynamics of incidence, prevalence, and DALYs, offering a critical basis for optimizing prevention and control strategies and improving public health interventions. Future interdisciplinary research and strengthened international cooperation are needed to address the global health challenge of musculoskeletal disorders.

This study systematically integrated data from GBD 1990 to 2021, an authoritative global health resource, to construct the first multidimensional assessment framework for musculoskeletal disorders in postmenopausal women. Its comparative analysis across regions and over time provides evidence-based guidance for precise prevention and treatment strategies. Epidemiologic analysis of postmenopausal women with musculoskeletal disorders offers valuable insights for disease management.

Our study has several limitations. First, the GBD 2021 database lacks reliable epidemiological data from low- and middle-income countries, meaning estimates may not be fully accurate. Second, rheumatoid arthritis, osteoarthritis, gout, neck pain, and low back pain are not differentiated in the GBD database; thus, detailed subtype analyses were not possible. Third, the data are aggregated at the national level and do not reflect subnational, ethnic, or socioeconomic variations.

## 5. Conclusion

Analysis of the GBD database identified an epidemiological pattern of “incidence-prevalence/DALYs separation” in musculoskeletal disorders. This suggests that widespread early screening and improved interventions have reduced new cases, while population aging has increased the accumulation of existing cases. Although improvements in chronic disease management have reduced individual disability severity, the overall disease duration has been significantly prolonged. Therefore, future prevention and control strategies should shift from solely reducing incidence to a comprehensive management cycle encompassing “prevention-treatment-rehabilitation,” emphasizing long-term functional maintenance for older populations. Globally, countries should strengthen disease surveillance and patient care for postmenopausal women.

## Author contributions

**Data curation:** Jingkai Di.

**Methodology:** Zijian Guo.

**Validation:** Yingda Qin.

**Supervision:** Zehui Yan.

**Funding acquisition:** Chuan Xiang.

**Writing – original draft:** Zui Tian, Changjiang Mu.

**Writing – review & editing:** Chuan Xiang.


