# Supplementary material for: Global, regional, and national burdens of musculoskeletal disorders in postmenopausal women from 1990 to 2021 and projections to 2050: A population-based observational study using GBD 2021 data
Source: Medicine (Baltimore). 2026 Jul 3;105(27):e49611. doi: 10.1097/MD.0000000000049611 (PMC13336975; doi:10.1097/MD.0000000000049611)
Supplement: Supplementary file 1 [file medi-105-e49611-s001.docx]

| Supplementary Table 1: Age-standardized musculoskeletal disease burden outcomes for menopausal women in 204 countries and territories. | | | | | | | | | |
| --- | --- | --- | --- | --- | --- | --- | --- | --- | --- |
| location | Incidence | | | Prevalence | | | DALYs | | |
|  | 1990 (per 100,000 population, 95% UI) | 2021 (per 100,000 population, 95% UI) | EAPCs (95% CI) | 1990 (per 100,000 population, 95% UI) | 2021 (per 100,000 population, 95% UI) | EAPCs (95% CI) | 1990 (per 100,000 population, 95% UI) | 2021 (per 100,000 population, 95% UI) | EAPCs (95% CI) |
| Afghanistan | 11295.28(8487.59,14677.46) | 11509.70(8684.57,14739.00) | 0.06(0.05,0.07) | 48281.13(42002.68,55004.08) | 51644.91(45917.42,57926.39) | 0.26(0.24,0.28) | 4528.11(2992.38,6426.47) | 4824.04(3281.65,6802.37) | 0.27(0.24,0.31) |
| Algeria | 11437.77(8598.25,14730.92) | 11660.13(8890.66,14996.96) | 0.07(0.05,0.08) | 53609.51(48012.05,59651.38) | 58677.00(53277.36,64318.98) | 0.30(0.29,0.31) | 5086.46(3458.15,7231.84) | 5556.56(3815.16,7837.64) | 0.30(0.28,0.31) |
| American Samoa | 11266.51(8499.43,14476.90) | 11349.65(8632.17,14591.31) | 0.01(0.00,0.02) | 57514.93(52496.60,62894.75) | 59710.30(54757.24,64907.37) | 0.10(0.09,0.12) | 5187.31(3556.14,7438.74) | 5232.32(3576.69,7505.95) | 0.02(0.00,0.03) |
| Andorra | 13046.40(9846.70,16866.20) | 13060.95(9886.65,16859.85) | 0.01(-0.01,0.03) | 57624.70(52099.86,63510.42) | 58488.38(53067.38,64228.77) | 0.05(0.03,0.06) | 5232.98(3535.20,7580.44) | 5182.45(3472.16,7612.68) | -0.03(-0.04,-0.01) |
| Angola | 11576.22(8688.85,14987.56) | 11320.00(8540.84,14564.66) | -0.08(-0.10,-0.06) | 54452.56(49029.76,60183.41) | 56033.85(50758.91,61500.66) | 0.09(0.09,0.09) | 4891.13(3339.64,7049.09) | 5001.66(3419.64,7199.32) | 0.08(0.07,0.08) |
| Antigua and Barbuda | 9472.80(7196.40,12167.17) | 9640.21(7354.26,12250.88) | 0.07(0.06,0.07) | 53870.28(49033.49,58741.22) | 56613.12(51658.41,61625.41) | 0.16(0.15,0.16) | 4646.20(3192.60,6703.17) | 4961.97(3435.67,7086.89) | 0.21(0.20,0.22) |
| Argentina | 13138.37(9944.91,16861.41) | 13485.61(10150.17,17367.11) | 0.08(0.03,0.12) | 65865.05(60517.47,71689.34) | 69107.85(63720.19,74577.30) | 0.14(0.11,0.16) | 6747.29(4735.42,9569.08) | 7229.97(5102.16,10095.22) | 0.20(0.16,0.24) |
| Armenia | 12925.11(9708.87,16797.82) | 13167.31(9821.69,17075.28) | 0.07(0.06,0.08) | 55317.91(49219.93,61934.86) | 61580.86(55585.17,67803.02) | 0.41(0.39,0.43) | 5101.30(3412.86,7397.43) | 5699.32(3852.10,8190.74) | 0.44(0.42,0.46) |
| Australia | 14342.48(10883.25,18357.10) | 14617.25(11059.82,18630.61) | 0.07(0.03,0.10) | 65557.51(60254.76,70949.52) | 68320.63(63007.02,73853.76) | 0.13(0.11,0.15) | 6815.58(4834.06,9481.14) | 7077.23(4966.47,9985.13) | 0.10(0.06,0.14) |
| Austria | 12651.34(9656.73,16229.24) | 12616.94(9573.63,16212.18) | 0.03(-0.01,0.07) | 57887.55(52725.49,63237.98) | 59462.23(54327.67,64752.18) | 0.08(0.04,0.12) | 5497.14(3794.06,7814.14) | 5560.11(3806.17,8067.15) | 0.04(-0.04,0.12) |
| Azerbaijan | 12658.88(9437.73,16455.11) | 12885.85(9669.68,16618.36) | 0.08(0.06,0.10) | 58117.06(52016.08,64346.81) | 61718.90(55757.78,67780.27) | 0.25(0.20,0.30) | 5316.72(3603.08,7663.68) | 5597.18(3789.25,8124.14) | 0.24(0.20,0.29) |
| Bahamas | 9538.28(7234.07,12267.34) | 9676.90(7411.45,12353.50) | 0.04(0.03,0.05) | 54939.44(49962.52,59825.22) | 57190.93(52359.34,62202.87) | 0.14(0.13,0.14) | 4840.03(3345.71,6916.11) | 5072.15(3517.75,7192.60) | 0.17(0.15,0.18) |
| Bahrain | 11605.15(8754.34,15003.63) | 11787.05(8954.22,15091.25) | 0.06(0.05,0.06) | 57695.93(52333.26,63371.29) | 61120.47(55880.54,66630.20) | 0.20(0.20,0.21) | 5539.46(3848.19,7758.96) | 5963.43(4161.65,8233.15) | 0.26(0.24,0.28) |
| Bangladesh | 12879.60(9625.01,16679.07) | 12499.37(9318.60,16271.16) | -0.06(-0.10,-0.03) | 64244.42(58625.15,70260.39) | 66332.98(60757.72,72192.68) | 0.14(0.11,0.16) | 6994.78(5050.44,9533.36) | 7117.81(5119.68,9795.52) | 0.11(0.06,0.15) |
| Barbados | 9614.69(7282.36,12324.46) | 9749.25(7456.88,12433.90) | 0.05(0.04,0.06) | 54831.76(50097.52,59862.07) | 57389.55(52710.41,62200.84) | 0.14(0.14,0.15) | 4944.95(3440.17,7039.29) | 5170.71(3610.45,7353.59) | 0.16(0.14,0.19) |
| Belarus | 13845.71(10431.30,17904.37) | 13979.88(10512.26,18003.03) | 0.03(0.02,0.04) | 59089.69(52947.83,65759.63) | 62310.46(56385.60,68429.40) | 0.20(0.18,0.21) | 5611.45(3803.66,8071.28) | 5893.87(4035.79,8452.74) | 0.20(0.17,0.23) |
| Belgium | 13078.19(9847.13,16873.96) | 13165.12(10041.43,16756.53) | 0.02(0.00,0.05) | 57840.06(52363.09,63720.02) | 58678.42(53217.17,64281.54) | 0.04(0.03,0.04) | 5435.07(3719.45,7781.84) | 5374.50(3650.03,7725.07) | -0.08(-0.10,-0.05) |
| Belize | 9605.72(7283.62,12369.94) | 9849.49(7511.58,12595.15) | 0.08(0.07,0.09) | 52203.77(47421.05,57094.72) | 56307.27(51419.09,61247.69) | 0.24(0.22,0.25) | 4524.05(3079.32,6493.46) | 4923.67(3387.08,7058.37) | 0.27(0.25,0.29) |
| Benin | 11022.85(8205.82,14223.22) | 10927.80(8300.15,14142.87) | -0.03(-0.06,0.00) | 50738.84(45486.78,56515.59) | 53808.28(48585.46,59309.33) | 0.19(0.18,0.20) | 4617.35(3190.48,6525.26) | 4828.22(3315.91,6807.67) | 0.14(0.12,0.16) |
| Bermuda | 9789.23(7479.40,12512.13) | 9892.26(7549.17,12604.97) | 0.02(0.01,0.03) | 56377.55(51504.39,61383.84) | 58424.30(53511.70,63434.98) | 0.12(0.11,0.12) | 4969.85(3432.46,7125.20) | 5140.02(3543.98,7379.52) | 0.11(0.10,0.12) |
| Bhutan | 12382.76(9255.02,16101.16) | 12505.44(9347.06,16170.96) | 0.02(0.01,0.04) | 59002.34(53294.18,65338.27) | 62506.85(57049.15,68543.17) | 0.20(0.19,0.20) | 6217.34(4460.40,8584.42) | 6591.07(4658.94,9125.18) | 0.20(0.20,0.21) |
| Bolivia (Plurinational State of) | 9380.55(7171.54,12003.13) | 9694.90(7384.19,12423.98) | 0.11(0.08,0.13) | 54037.78(49189.17,59186.64) | 58083.96(53163.47,63029.83) | 0.23(0.22,0.23) | 4816.07(3328.58,6806.23) | 5219.78(3657.66,7355.39) | 0.26(0.24,0.27) |
| Bosnia and Herzegovina | 13755.96(10350.49,17713.31) | 13882.81(10386.02,17991.04) | 0.04(0.03,0.05) | 55542.07(49354.47,62239.27) | 59723.14(53678.83,66324.76) | 0.29(0.27,0.32) | 5457.34(3733.22,7723.49) | 5796.65(3967.01,8300.95) | 0.28(0.26,0.31) |
| Botswana | 10859.55(8174.24,14087.68) | 11010.66(8332.38,14220.91) | 0.05(0.04,0.06) | 51730.70(46604.52,57336.25) | 55832.95(50736.36,61115.75) | 0.24(0.23,0.24) | 4779.25(3276.87,6747.12) | 5014.47(3466.77,7112.77) | 0.18(0.16,0.19) |
| Brazil | 11973.29(9098.13,15348.87) | 12638.18(9663.12,16071.05) | 0.16(0.12,0.19) | 60831.53(55518.30,66380.90) | 63530.75(58327.78,68864.79) | 0.11(0.09,0.13) | 5870.18(4106.62,8242.23) | 6151.50(4299.25,8622.57) | 0.09(0.05,0.14) |
| Brunei Darussalam | 12404.18(9461.33,15774.43) | 12224.17(9335.86,15600.55) | -0.05(-0.08,-0.02) | 66305.66(61016.60,71456.03) | 68648.19(63716.35,73784.07) | 0.11(0.09,0.12) | 6350.68(4429.06,9103.97) | 6513.59(4532.36,9347.70) | 0.09(0.05,0.12) |
| Bulgaria | 13909.12(10357.19,17841.51) | 13947.79(10472.90,17934.27) | 0.01(0.00,0.01) | 58574.84(52181.31,65098.75) | 60954.61(54755.75,67390.54) | 0.14(0.13,0.14) | 5634.31(3842.55,8063.56) | 5825.03(3946.51,8352.88) | 0.13(0.12,0.14) |
| Burkina Faso | 11030.61(8320.20,14173.87) | 10841.31(8116.02,13993.87) | -0.08(-0.10,-0.05) | 48998.30(43868.40,54582.95) | 50491.49(45361.31,56174.70) | 0.09(0.08,0.10) | 4530.09(3125.19,6364.66) | 4652.73(3182.65,6608.76) | 0.09(0.07,0.11) |
| Burundi | 11849.88(8872.79,15257.34) | 11619.90(8669.59,15100.43) | -0.08(-0.09,-0.07) | 50837.81(45326.97,56989.39) | 49072.82(43338.40,55337.14) | -0.13(-0.18,-0.08) | 4770.36(3261.65,6769.90) | 4474.36(3027.42,6509.93) | -0.23(-0.31,-0.16) |
| Cabo Verde | 10657.65(8047.36,13690.21) | 10591.29(8010.13,13633.99) | -0.02(-0.05,-0.00) | 50094.35(44881.28,55625.34) | 53899.77(48797.37,59142.64) | 0.25(0.24,0.26) | 4577.88(3116.94,6462.61) | 4794.67(3272.53,6818.48) | 0.16(0.15,0.17) |
| Cambodia | 10326.83(7745.12,13418.95) | 10091.18(7564.50,13109.42) | -0.07(-0.08,-0.07) | 47070.31(41667.17,53219.82) | 49476.96(44377.92,55293.16) | 0.17(0.16,0.17) | 4374.76(3013.43,6140.00) | 4521.40(3119.47,6381.11) | 0.11(0.10,0.12) |
| Cameroon | 11304.85(8457.95,14594.19) | 11115.53(8409.98,14364.89) | -0.06(-0.09,-0.03) | 52291.04(47017.38,58040.11) | 54551.99(49406.19,60003.53) | 0.13(0.12,0.13) | 4831.44(3349.82,6815.62) | 4972.55(3424.01,7071.79) | 0.09(0.08,0.10) |
| Canada | 11369.59(8518.73,14742.82) | 10932.89(8296.83,14158.90) | -0.07(-0.11,-0.02) | 59726.87(54033.70,65654.99) | 61372.82(55875.55,67197.56) | 0.07(0.04,0.10) | 6082.84(4298.89,8513.08) | 6107.38(4316.62,8589.65) | 0.01(-0.03,0.05) |
| Central African Republic | 11289.58(8444.70,14527.48) | 11184.48(8362.92,14525.48) | -0.04(-0.05,-0.03) | 50732.64(45346.06,56554.25) | 51215.06(46015.16,56823.91) | 0.01(0.01,0.02) | 4646.28(3222.70,6551.88) | 4677.00(3239.89,6648.39) | 0.00(-0.00,0.01) |
| Chad | 11429.26(8591.61,14674.27) | 11233.18(8433.84,14485.95) | -0.06(-0.10,-0.02) | 50404.83(45097.67,56101.90) | 51282.51(45884.93,57248.71) | 0.06(0.05,0.07) | 4719.75(3236.19,6643.00) | 4762.83(3293.82,6680.88) | 0.04(0.02,0.07) |
| Chile | 13244.35(9946.29,17039.18) | 13706.77(10371.84,17616.32) | 0.08(0.04,0.12) | 68348.84(62764.66,73780.63) | 69156.26(63941.74,74510.06) | -0.04(-0.10,0.03) | 7375.28(5227.36,10194.20) | 7283.11(5155.30,10105.58) | -0.17(-0.28,-0.05) |
| China | 11846.68(8958.64,15176.44) | 10669.01(8226.76,13515.98) | -0.15(-0.23,-0.07) | 55166.73(49907.05,60838.72) | 56834.92(51900.65,61923.69) | 0.27(0.20,0.33) | 5096.44(3490.87,7284.25) | 4989.92(3432.06,7125.26) | 0.14(0.07,0.22) |
| Colombia | 10704.76(8031.57,13777.78) | 10675.62(8105.39,13669.78) | 0.02(0.01,0.03) | 58074.52(52804.56,63599.50) | 61708.77(56624.87,67013.49) | 0.22(0.21,0.23) | 5621.91(3932.88,7854.51) | 6038.29(4264.84,8440.46) | 0.29(0.27,0.31) |
| Comoros | 11438.81(8669.92,14711.24) | 11531.74(8650.97,14947.20) | -0.00(-0.02,0.01) | 50400.30(45257.62,56139.71) | 52839.98(47536.74,58494.95) | 0.14(0.14,0.15) | 4644.83(3220.39,6532.15) | 4848.96(3328.66,6925.12) | 0.12(0.11,0.13) |
| Congo | 11029.24(8326.75,14187.54) | 11120.19(8401.90,14275.89) | 0.01(-0.00,0.03) | 53118.37(47956.64,58654.09) | 55310.90(49956.26,60641.69) | 0.12(0.10,0.15) | 4833.74(3337.55,6889.36) | 4966.08(3438.34,7089.97) | 0.07(0.04,0.11) |
| Cook Islands | 11142.72(8443.33,14352.80) | 11365.75(8640.90,14592.40) | 0.09(0.08,0.10) | 56303.91(51277.78,61550.83) | 60027.42(55081.51,65243.59) | 0.20(0.19,0.21) | 5003.98(3396.74,7154.82) | 5256.00(3606.66,7544.28) | 0.17(0.16,0.18) |
| Costa Rica | 10557.00(7914.08,13604.41) | 10480.80(7932.08,13451.89) | -0.02(-0.03,-0.02) | 58093.45(52852.60,63588.60) | 61475.13(56468.46,66869.11) | 0.18(0.17,0.19) | 5596.68(3910.22,7878.49) | 5972.74(4211.32,8362.98) | 0.21(0.19,0.22) |
| Cote d'Ivoire | 11261.19(8560.80,14420.05) | 11081.50(8319.31,14359.42) | -0.04(-0.07,-0.01) | 51709.14(46594.56,57211.03) | 53663.54(48450.70,59273.28) | 0.12(0.11,0.12) | 4679.13(3220.46,6538.14) | 4887.90(3366.96,6958.59) | 0.14(0.11,0.16) |
| Croatia | 13267.25(10091.62,16942.44) | 13867.29(10364.31,17894.14) | 0.17(0.11,0.24) | 56100.24(50432.05,62132.53) | 60086.64(54150.31,66446.70) | 0.27(0.22,0.32) | 5296.25(3662.40,7507.27) | 5820.31(3977.97,8337.05) | 0.38(0.29,0.46) |
| Cuba | 9574.78(7249.18,12234.88) | 9582.56(7498.83,11825.01) | 0.06(0.04,0.08) | 51322.73(46610.61,56061.39) | 53842.19(49293.00,58278.55) | 0.19(0.17,0.21) | 4384.07(2982.59,6287.03) | 4559.06(3135.86,6521.59) | 0.19(0.16,0.22) |
| Cyprus | 12909.41(9695.25,16579.10) | 13076.48(9907.46,16788.43) | 0.04(0.02,0.06) | 56924.30(51568.64,62680.53) | 59964.90(54766.10,65539.72) | 0.17(0.16,0.19) | 5742.10(4024.47,8135.64) | 5867.84(4048.60,8296.88) | 0.05(0.04,0.07) |
| Czechia | 14345.72(10713.95,18624.40) | 14343.88(10739.85,18463.51) | -0.01(-0.02,0.01) | 60151.46(53853.96,66786.09) | 62661.28(56535.30,69201.49) | 0.14(0.13,0.14) | 5766.64(3943.59,8331.62) | 6090.89(4152.46,8694.57) | 0.18(0.18,0.19) |
| Democratic People's Republic of Korea | 11543.71(8678.50,14939.31) | 11345.46(8593.53,14663.31) | -0.07(-0.08,-0.06) | 54879.70(49450.80,60698.67) | 56735.46(51561.91,62357.16) | 0.10(0.10,0.11) | 5240.08(3590.68,7412.11) | 5376.24(3712.56,7628.82) | 0.08(0.08,0.09) |
| Democratic Republic of the Congo | 11427.15(8589.74,14857.51) | 11229.13(8480.80,14493.20) | -0.07(-0.08,-0.06) | 52155.37(46747.31,57983.67) | 52132.64(46894.37,57947.87) | -0.03(-0.06,-0.00) | 4760.37(3271.71,6759.66) | 4763.80(3301.30,6810.37) | -0.02(-0.04,0.00) |
| Denmark | 13963.51(10219.13,18272.08) | 13105.26(9288.87,17793.50) | -0.33(-0.42,-0.25) | 64324.55(59328.66,69217.17) | 62503.23(56383.10,68979.75) | -0.15(-0.19,-0.10) | 6668.94(4702.97,9337.14) | 6428.91(4378.74,9259.46) | -0.25(-0.32,-0.17) |
| Djibouti | 11412.83(8553.00,14755.23) | 11289.52(8431.51,14556.97) | -0.05(-0.07,-0.04) | 50541.36(45228.94,56350.27) | 53513.47(48285.70,58884.54) | 0.21(0.19,0.23) | 4648.48(3172.74,6627.74) | 4803.69(3264.28,6831.71) | 0.12(0.11,0.14) |
| Dominica | 9612.91(7309.76,12357.60) | 9753.27(7404.32,12493.02) | 0.03(0.02,0.04) | 52662.17(47832.83,57764.14) | 56046.10(51277.63,61071.50) | 0.20(0.19,0.21) | 4603.28(3172.92,6532.44) | 4864.27(3337.91,6926.75) | 0.18(0.17,0.19) |
| Dominican Republic | 9462.51(7257.52,12059.54) | 9787.76(7422.69,12553.19) | 0.11(0.10,0.12) | 52101.23(47244.34,57044.37) | 55829.44(50961.46,60860.97) | 0.24(0.23,0.24) | 4467.64(3038.90,6391.00) | 4766.84(3265.60,6913.05) | 0.24(0.21,0.26) |
| Ecuador | 9344.20(7187.32,11841.81) | 9383.61(7318.97,11770.35) | 0.04(-0.01,0.08) | 56307.32(51446.02,61400.69) | 59105.03(54436.91,63942.85) | 0.21(0.18,0.24) | 4973.93(3446.03,7085.58) | 5207.39(3607.06,7446.70) | 0.20(0.17,0.24) |
| Egypt | 11462.71(8600.94,14735.95) | 11867.37(8963.00,15250.71) | 0.11(0.09,0.13) | 53756.11(48283.04,59604.57) | 58582.75(53173.07,64218.70) | 0.25(0.24,0.27) | 5120.85(3501.94,7245.04) | 5594.66(3857.73,7832.78) | 0.28(0.25,0.30) |
| El Salvador | 10346.38(7811.20,13231.04) | 10480.94(7930.05,13428.11) | 0.06(0.05,0.07) | 55664.24(50363.41,61312.25) | 60056.80(54894.46,65464.85) | 0.26(0.24,0.27) | 5121.40(3499.98,7292.87) | 5592.38(3885.50,7911.12) | 0.31(0.30,0.33) |
| Equatorial Guinea | 11199.68(8429.16,14470.79) | 11241.40(8519.39,14467.25) | 0.02(0.01,0.04) | 49949.81(44578.47,55741.60) | 56480.60(51305.32,61836.88) | 0.48(0.45,0.51) | 4587.09(3186.61,6475.26) | 5092.55(3507.42,7254.80) | 0.41(0.38,0.44) |
| Eritrea | 10919.61(8161.43,14247.93) | 11005.06(8235.62,14329.84) | 0.04(0.03,0.05) | 44154.17(38697.39,50387.92) | 48881.04(43288.35,54576.13) | 0.36(0.35,0.37) | 3802.11(2501.12,5510.60) | 4335.35(2923.51,6222.37) | 0.49(0.47,0.50) |
| Estonia | 13882.94(10510.36,17885.42) | 13973.92(10500.60,18153.92) | 0.03(0.02,0.04) | 59899.19(54102.17,66071.71) | 63983.67(58147.56,70214.23) | 0.25(0.23,0.26) | 5823.96(4027.17,8284.22) | 6348.17(4411.35,9070.28) | 0.33(0.31,0.36) |
| Eswatini | 10574.94(8046.56,13511.16) | 10538.12(8004.91,13394.44) | -0.03(-0.04,-0.01) | 50943.84(46125.35,56194.89) | 54358.14(49466.23,59290.73) | 0.19(0.18,0.20) | 4692.72(3270.23,6643.22) | 4879.59(3391.60,6862.90) | 0.11(0.08,0.14) |
| Ethiopia | 12679.35(9554.98,16315.14) | 12170.33(9216.73,15584.79) | -0.13(-0.13,-0.12) | 52292.54(46783.51,58520.68) | 55294.56(49966.22,60927.83) | 0.22(0.20,0.24) | 4848.71(3346.62,6857.15) | 4949.90(3401.37,7041.65) | 0.11(0.08,0.14) |
| Fiji | 11024.07(8327.62,14308.95) | 11041.71(8375.99,14211.69) | 0.02(0.01,0.03) | 54472.77(49307.12,60063.07) | 57988.64(53056.76,63265.94) | 0.21(0.20,0.21) | 4867.31(3339.98,6923.33) | 5041.43(3446.51,7120.89) | 0.13(0.13,0.14) |
| Finland | 12383.80(9469.80,15819.27) | 12382.35(9404.62,15952.84) | 0.01(-0.01,0.03) | 56247.28(51094.41,61445.94) | 58297.22(53067.99,63763.08) | 0.13(0.11,0.15) | 5346.32(3745.96,7579.72) | 5382.30(3710.36,7722.24) | 0.04(-0.01,0.09) |
| France | 12916.22(9825.72,16554.96) | 13271.03(10047.20,17050.01) | 0.08(0.05,0.11) | 56772.00(51519.66,62508.99) | 59144.47(53545.74,64873.67) | 0.12(0.11,0.13) | 5248.55(3630.56,7469.16) | 5520.14(3783.26,7985.82) | 0.14(0.11,0.17) |
| Gabon | 11018.34(8235.74,14289.74) | 11123.38(8384.38,14288.58) | 0.03(0.02,0.04) | 52024.64(46661.53,57510.40) | 55981.15(50889.55,61371.49) | 0.23(0.22,0.24) | 4746.77(3278.18,6689.76) | 5053.01(3506.94,7184.76) | 0.20(0.19,0.22) |
| Gambia | 10685.90(8035.34,13797.39) | 10588.31(7996.22,13748.59) | -0.03(-0.06,-0.00) | 50336.71(45260.38,55786.40) | 53286.60(48221.36,58489.89) | 0.19(0.18,0.20) | 4523.37(3115.38,6422.71) | 4683.12(3226.86,6652.22) | 0.12(0.11,0.14) |
| Georgia | 12355.61(9242.40,15938.53) | 12326.10(9290.49,15938.78) | -0.07(-0.09,-0.05) | 55850.83(50211.34,61733.33) | 57913.83(51960.98,63942.06) | 0.06(0.01,0.11) | 5070.89(3448.14,7322.07) | 5134.35(3494.58,7426.01) | -0.01(-0.05,0.03) |
| Germany | 14111.37(10717.89,18062.02) | 14061.80(10697.19,17968.50) | 0.01(-0.02,0.03) | 61476.58(55787.47,67488.40) | 62454.36(57039.76,68141.12) | 0.06(0.04,0.07) | 6087.39(4187.38,8610.68) | 6093.04(4197.25,8677.99) | 0.03(0.00,0.05) |
| Ghana | 10368.40(7885.23,13137.22) | 10065.70(7834.81,12626.43) | -0.09(-0.11,-0.08) | 52859.48(47793.00,57975.55) | 53722.05(49059.32,58601.37) | 0.07(0.03,0.12) | 4585.73(3135.19,6602.49) | 4660.09(3193.36,6667.93) | 0.07(0.04,0.10) |
| Global | 12561.68(9570.39,16041.15) | 11951.59(9206.61,15124.70) | -0.12(-0.14,-0.10) | 58444.23(53332.73,63929.04) | 60482.07(55619.92,65532.64) | 0.15(0.13,0.17) | 5620.67(3918.54,7929.81) | 5722.74(4007.39,8045.78) | 0.11(0.09,0.13) |
| Greece | 12636.54(9519.25,16177.43) | 12914.12(9807.50,16592.52) | 0.07(0.04,0.10) | 56796.81(51423.51,62441.03) | 60239.25(54913.15,65553.26) | 0.27(0.23,0.32) | 5279.97(3592.68,7585.07) | 5774.98(3968.93,8240.67) | 0.33(0.29,0.38) |
| Greenland | 11117.45(8320.35,14400.08) | 10890.12(8172.69,14048.10) | -0.01(-0.04,0.03) | 56201.47(50430.63,62300.05) | 59789.70(54071.60,65639.38) | 0.23(0.22,0.24) | 5309.60(3696.73,7542.22) | 5658.57(3875.24,8041.96) | 0.27(0.25,0.30) |
| Grenada | 9483.45(7203.00,12153.74) | 9716.84(7379.56,12412.21) | 0.07(0.06,0.08) | 51654.66(46866.30,56712.61) | 55670.60(50694.80,60768.45) | 0.23(0.22,0.25) | 4502.35(3100.55,6431.74) | 4866.92(3385.45,6955.98) | 0.26(0.25,0.28) |
| Guam | 11089.23(8366.11,14271.89) | 11181.13(8466.73,14344.94) | 0.04(0.03,0.06) | 56484.16(51443.04,61724.35) | 59471.44(54431.29,64595.40) | 0.18(0.17,0.19) | 5197.35(3564.44,7341.16) | 5359.71(3661.64,7673.75) | 0.13(0.12,0.14) |
| Guatemala | 10942.58(8228.42,14025.69) | 10904.21(8191.80,14034.45) | -0.01(-0.02,0.01) | 56652.51(51494.70,62351.70) | 59495.48(54212.82,65263.84) | 0.17(0.16,0.18) | 5413.58(3798.73,7630.11) | 5672.49(3974.65,7971.51) | 0.19(0.17,0.21) |
| Guinea | 10927.09(8181.85,14099.23) | 10935.29(8251.60,14189.25) | -0.02(-0.05,0.01) | 49548.60(44320.09,55295.62) | 51484.98(46313.30,57081.02) | 0.11(0.10,0.11) | 4556.88(3160.30,6449.88) | 4723.27(3235.66,6658.93) | 0.10(0.08,0.12) |
| Guinea-Bissau | 10725.62(8053.01,13886.33) | 10665.82(8068.72,13801.10) | -0.04(-0.06,-0.01) | 49386.13(44272.96,55157.67) | 51229.04(46043.60,56658.34) | 0.11(0.10,0.11) | 4499.76(3104.57,6396.15) | 4648.85(3208.31,6568.24) | 0.09(0.08,0.10) |
| Guyana | 9474.98(7186.19,12169.30) | 9646.62(7359.12,12311.26) | 0.05(0.04,0.06) | 51319.71(46284.71,56400.45) | 54892.62(49949.54,60023.40) | 0.22(0.21,0.23) | 4258.78(2892.03,6115.27) | 4563.95(3144.37,6556.49) | 0.23(0.22,0.25) |
| Haiti | 9328.82(7070.00,11979.08) | 9430.25(7144.80,12133.50) | 0.02(0.01,0.03) | 47999.78(43138.70,53024.87) | 49936.91(45054.22,55126.97) | 0.10(0.09,0.12) | 4248.17(2966.27,6020.32) | 4319.76(3005.93,6118.78) | 0.01(-0.03,0.06) |
| Honduras | 10361.91(7826.40,13361.50) | 10463.50(7925.48,13450.69) | 0.04(0.03,0.05) | 56263.89(51150.88,61769.64) | 59476.66(54356.89,64809.03) | 0.19(0.18,0.19) | 5460.82(3832.94,7592.89) | 5908.77(4240.20,8207.32) | 0.28(0.27,0.29) |
| Hungary | 14440.80(10878.81,18656.70) | 14520.63(10934.49,18752.89) | 0.00(-0.01,0.01) | 60575.04(54068.98,67586.00) | 63321.74(57095.20,69916.68) | 0.15(0.15,0.16) | 5967.60(4101.72,8449.53) | 6337.80(4381.68,9007.88) | 0.23(0.22,0.25) |
| Iceland | 13483.27(10205.96,17282.97) | 13306.99(10062.55,17016.44) | -0.06(-0.09,-0.04) | 60933.99(55686.53,66545.64) | 61752.10(56627.10,67235.84) | 0.04(0.02,0.06) | 5896.86(4071.29,8483.91) | 5963.06(4095.83,8532.12) | 0.04(0.02,0.06) |
| India | 12423.62(9373.10,15984.37) | 11613.91(8798.47,14877.24) | -0.26(-0.36,-0.16) | 57990.88(52618.68,63904.12) | 61711.37(56413.68,67347.76) | 0.19(0.14,0.24) | 5613.27(3995.64,7798.45) | 5968.65(4244.38,8278.35) | 0.18(0.09,0.27) |
| Indonesia | 11258.87(8501.74,14500.15) | 11291.02(8557.57,14468.30) | 0.04(0.02,0.05) | 50660.37(45346.52,56465.00) | 54517.30(49409.96,60151.75) | 0.25(0.23,0.26) | 4713.55(3233.92,6655.70) | 5038.60(3457.53,7115.07) | 0.25(0.23,0.26) |
| Iran (Islamic Republic of) | 13281.44(10094.36,17091.85) | 13176.16(10077.42,16875.35) | -0.03(-0.03,-0.02) | 56302.36(50318.52,62763.26) | 60144.93(54467.32,65957.21) | 0.23(0.21,0.25) | 5531.97(3772.41,7829.24) | 5887.10(4063.91,8274.61) | 0.24(0.22,0.25) |
| Iraq | 11577.61(8794.82,14873.21) | 11636.38(8810.93,14995.13) | 0.02(0.01,0.03) | 52675.72(46879.39,58653.92) | 56915.54(51313.14,62671.27) | 0.22(0.21,0.23) | 4833.97(3253.71,6888.96) | 5275.18(3575.19,7458.41) | 0.26(0.24,0.27) |
| Ireland | 13156.03(9888.02,16882.64) | 13327.48(10197.91,16956.35) | 0.02(-0.01,0.04) | 59231.18(53727.29,64990.19) | 61319.96(56066.17,66634.35) | 0.11(0.10,0.12) | 5960.51(4137.54,8424.73) | 6094.30(4266.62,8560.12) | 0.07(0.06,0.08) |
| Israel | 13301.71(10128.14,17008.65) | 13253.39(10103.11,17142.24) | -0.03(-0.07,-0.00) | 59573.65(54264.73,65154.65) | 61070.37(55860.84,66548.33) | -0.08(-0.15,-0.01) | 5753.09(3969.42,8237.17) | 5860.16(4037.40,8393.30) | -0.07(-0.13,-0.01) |
| Italy | 13581.28(10345.41,17312.08) | 13672.69(10444.22,17436.81) | -0.02(-0.04,0.01) | 58457.85(53054.61,64069.12) | 61329.38(56042.51,66786.59) | 0.15(0.12,0.18) | 5474.84(3715.80,7833.67) | 5885.13(4035.39,8388.69) | 0.18(0.15,0.21) |
| Jamaica | 9633.15(7253.56,12490.36) | 9834.65(7495.20,12699.85) | 0.05(0.03,0.07) | 52663.32(47813.90,57801.40) | 55855.65(50987.59,60849.68) | 0.19(0.18,0.21) | 4693.17(3234.06,6715.45) | 5002.36(3469.88,7136.31) | 0.20(0.17,0.22) |
| Japan | 14113.40(10833.11,17936.68) | 13846.21(10630.44,17607.19) | -0.04(-0.08,0.01) | 67846.26(62753.57,73076.94) |  | 0.27(0.16,0.39) | 6963.74(4876.09,9867.53) | 7224.22(5032.84,10261.98) | 0.23(0.14,0.33) |
| Jordan | 11641.85(8818.31,15013.27) | 11873.26(8960.32,15314.89) | 0.06(0.06,0.07) | 56195.93(50743.76,62042.72) | 60340.68(55220.77,65885.95) | 0.24(0.23,0.25) | 5412.61(3754.53,7636.67) | 5764.07(4010.08,8071.82) | 0.23(0.22,0.25) |
| Kazakhstan | 13029.30(9859.84,16637.40) | 13114.15(9869.67,16901.65) | 0.02(0.01,0.03) | 58631.82(52787.40,64702.10) | 63976.91(57669.69,70147.52) | 0.30(0.27,0.32) | 5361.25(3654.79,7697.62) | 5760.98(3906.01,8343.69) | 0.24(0.22,0.26) |
| Kenya | 13591.89(10289.37,17368.57) | 13182.18(10009.70,16903.34) | -0.06(-0.09,-0.03) | 56282.18(50613.86,62471.64) | 58119.93(52629.99,63921.39) | 0.11(0.10,0.12) | 5373.04(3702.51,7611.06) | 5438.12(3742.54,7721.72) | 0.08(0.06,0.10) |
| Kiribati | 11003.76(8275.56,14191.55) | 11241.30(8519.19,14446.91) | 0.11(0.09,0.13) | 54440.32(49364.41,59807.29) | 57179.36(51970.65,62494.26) | 0.15(0.14,0.17) | 4789.16(3265.95,6836.18) | 5034.66(3455.85,7107.23) | 0.18(0.16,0.19) |
| Kuwait | 11662.45(8805.42,15138.94) | 11921.62(9050.18,15293.74) | 0.08(0.07,0.09) | 58908.27(53829.83,64357.03) | 62061.11(56727.86,67426.44) | 0.21(0.20,0.23) | 5643.83(3871.26,7925.23) | 5980.37(4135.44,8373.31) | 0.25(0.22,0.28) |
| Kyrgyzstan | 12836.39(9586.06,16697.19) | 12806.08(9622.26,16488.00) | -0.01(-0.02,0.00) | 57579.75(51535.82,63840.24) | 61142.32(55110.54,67191.06) | 0.24(0.20,0.28) | 5403.77(3710.26,7777.44) | 5789.24(3996.50,8316.08) | 0.32(0.27,0.38) |
| Lao People's Democratic Republic | 9929.14(7469.14,12745.97) | 9878.24(7397.57,12707.02) | -0.02(-0.03,-0.01) | 47259.82(42456.95,52636.81) | 50495.57(45458.36,55768.11) | 0.22(0.20,0.23) | 4390.86(3047.11,6147.73) | 4626.93(3180.67,6500.76) | 0.17(0.15,0.18) |
| Latvia | 13826.51(10381.53,17903.67) | 13875.84(10436.27,17937.00) | 0.01(-0.00,0.02) | 58641.14(52449.04,65347.93) | 62882.17(57082.54,69176.97) | 0.27(0.25,0.28) | 5600.19(3815.36,8094.07) | 6134.81(4237.56,8746.90) | 0.35(0.33,0.38) |
| Lebanon | 11386.24(8577.66,14651.85) | 11620.51(8798.24,14997.95) | 0.09(0.08,0.10) | 52684.65(47071.72,58748.36) | 58902.14(53508.47,64606.03) | 0.39(0.37,0.40) | 4921.85(3306.47,6997.39) | 5538.32(3784.98,7819.28) | 0.44(0.42,0.46) |
| Lesotho | 10797.81(8100.69,14011.35) | 10786.58(8114.86,13878.54) | -0.02(-0.04,-0.01) | 50053.28(44803.09,55626.50) | 53450.81(48532.47,58680.70) | 0.22(0.22,0.23) | 4641.65(3184.19,6554.92) | 4818.53(3347.45,6806.01) | 0.14(0.13,0.16) |
| Liberia | 10930.72(8207.52,14144.94) | 10812.50(8124.71,13894.38) | -0.02(-0.04,-0.01) | 50790.67(45541.91,56478.44) | 53003.91(47771.41,58344.29) | 0.20(0.17,0.22) | 4533.03(3141.07,6383.27) | 4661.11(3252.91,6590.30) | 0.16(0.13,0.20) |
| Libya | 11571.50(8750.86,14955.32) | 11729.60(8910.69,15052.34) | 0.03(0.03,0.04) | 55539.48(50283.87,61402.66) | 58460.27(53173.26,64133.39) | 0.16(0.14,0.19) | 5250.46(3592.51,7448.25) | 5471.04(3765.29,7737.85) | 0.13(0.10,0.17) |
| Lithuania | 13790.10(10281.93,17891.37) | 13832.50(10412.61,17840.99) | -0.00(-0.01,0.01) | 58710.85(52560.09,65233.31) | 62287.52(56335.51,68668.27) | 0.22(0.21,0.23) | 5674.89(3858.75,8128.20) | 6070.64(4213.34,8650.40) | 0.23(0.21,0.26) |
| Luxembourg | 13295.74(10045.06,17150.06) | 13557.81(10349.83,17214.07) | 0.03(0.00,0.06) | 58666.36(53255.00,64470.27) | 61016.42(55612.48,66531.03) | 0.10(0.09,0.12) | 5563.34(3779.33,8028.45) | 5837.07(4016.28,8313.39) | 0.11(0.08,0.14) |
| Madagascar | 11976.81(8895.68,15427.78) | 11513.19(8612.25,14886.61) | -0.14(-0.16,-0.12) | 51064.60(45419.91,57234.94) | 50868.67(45469.82,56882.73) | -0.02(-0.02,-0.02) | 4842.88(3327.21,6862.59) | 4747.95(3277.34,6754.31) | -0.06(-0.07,-0.06) |
| Malawi | 11534.11(8673.04,14895.06) | 11590.25(8726.08,15112.26) | -0.01(-0.03,0.01) | 50264.25(45136.13,56004.38) | 52039.31(46570.12,57866.44) | 0.11(0.11,0.12) | 4645.97(3227.25,6601.83) | 4790.52(3289.66,6785.52) | 0.11(0.10,0.12) |
| Malaysia | 9712.96(7443.86,12359.76) | 9955.07(7511.52,12747.57) | 0.06(0.05,0.08) | 50198.76(45455.07,55253.71) | 54437.51(49275.72,59705.38) | 0.25(0.24,0.26) | 4512.46(3145.79,6297.72) | 4888.68(3363.36,6877.00) | 0.24(0.22,0.25) |
| Maldives | 9718.91(7309.37,12605.64) | 9718.68(7344.03,12556.18) | -0.00(-0.01,0.00) | 49151.58(44177.13,54519.42) | 53407.38(48304.32,58753.03) | 0.26(0.25,0.27) | 4425.91(3049.41,6192.93) | 4730.74(3231.63,6654.13) | 0.20(0.19,0.21) |
| Mali | 10040.19(7545.80,12923.43) | 10365.77(7786.42,13390.94) | 0.07(0.05,0.09) | 47263.66(42342.45,52484.06) | 50142.26(45130.85,55410.62) | 0.19(0.18,0.20) | 4179.85(2855.75,5901.72) | 4437.70(3050.61,6313.78) | 0.19(0.18,0.21) |
| Malta | 13311.13(10105.74,17202.93) | 13689.22(10493.77,17316.06) | 0.05(0.02,0.08) | 58676.34(53198.31,64444.46) | 61574.73(56443.77,66823.08) | 0.11(0.09,0.13) | 5608.33(3832.07,8083.90) | 5969.15(4112.99,8463.06) | 0.11(0.07,0.15) |
| Marshall Islands | 10821.09(8130.93,13918.68) | 10873.26(8242.57,13993.38) | 0.03(0.02,0.04) | 53555.92(48549.53,58911.50) | 56524.95(51601.78,61667.57) | 0.17(0.16,0.17) | 4743.27(3261.78,6759.69) | 4866.32(3336.08,6980.22) | 0.07(0.07,0.08) |
| Mauritania | 10458.30(7969.02,13321.67) | 10665.82(8026.30,13822.85) | 0.02(-0.01,0.05) | 50077.65(45068.68,55461.08) | 53731.15(48707.23,59011.02) | 0.20(0.19,0.22) | 4502.40(3147.48,6418.42) | 4829.57(3330.68,6907.84) | 0.19(0.17,0.22) |
| Mauritius | 10190.42(7696.98,13069.46) | 10136.43(7659.58,13109.68) | 0.01(-0.01,0.03) | 52613.91(47753.84,57786.63) | 55623.04(50583.14,60984.90) | 0.19(0.18,0.20) | 4794.62(3331.38,6730.32) | 5080.31(3556.00,7124.47) | 0.19(0.18,0.20) |
| Mexico | 10750.60(8196.96,13774.77) | 10657.60(8150.32,13571.35) | 0.01(-0.06,0.08) | 59544.88(54422.58,64970.30) | 63186.46(58275.19,68227.33) | 0.20(0.19,0.21) | 5863.35(4180.65,8145.25) | 6165.82(4372.58,8589.65) | 0.16(0.13,0.20) |
| Micronesia (Federated States of) | 11069.50(8351.09,14218.92) | 11297.25(8537.30,14550.63) | 0.08(0.06,0.09) | 54172.37(49166.23,59498.83) | 57453.31(52494.51,62654.83) | 0.19(0.17,0.21) | 4873.62(3354.03,6891.90) | 5060.12(3470.49,7187.00) | 0.13(0.11,0.14) |
| Monaco | 13021.04(9892.64,16733.07) | 13025.31(9915.64,16788.10) | -0.00(-0.02,0.02) | 60190.03(54758.29,65748.98) | 61168.31(55718.24,66636.08) | 0.05(0.04,0.06) | 5713.30(3866.14,8223.01) | 5753.63(3923.26,8260.58) | 0.02(0.01,0.04) |
| Mongolia | 12699.47(9437.14,16403.69) | 12738.02(9587.51,16469.97) | -0.02(-0.03,-0.01) | 55976.98(50141.52,62029.78) | 62147.75(55772.98,68420.43) | 0.37(0.35,0.40) | 5174.94(3501.78,7448.66) | 5636.16(3839.40,8211.14) | 0.32(0.30,0.34) |
| Montenegro | 13981.44(10401.51,18018.93) | 14142.18(10616.53,18235.52) | 0.03(0.02,0.04) | 59130.73(52949.53,65767.63) | 61571.20(55391.61,68285.27) | 0.16(0.15,0.17) | 5698.04(3875.41,8156.39) | 5939.42(4056.55,8487.84) | 0.18(0.16,0.20) |
| Morocco | 12166.70(9218.94,15564.09) | 12275.97(9256.87,15767.86) | 0.05(0.01,0.09) | 55838.11(50071.74,61815.26) | 58435.70(53021.11,64166.46) | 0.15(0.14,0.16) | 5454.14(3742.05,7689.00) | 5659.62(3935.07,7912.89) | 0.15(0.11,0.19) |
| Mozambique | 11871.43(8936.99,15424.38) | 11870.11(8813.65,15451.55) | 0.00(-0.00,0.01) | 49810.92(44072.85,56182.26) | 52149.26(46671.17,58436.72) | 0.15(0.14,0.16) | 4571.73(3119.47,6544.57) | 4796.00(3319.79,6833.97) | 0.19(0.17,0.21) |
| Myanmar | 9092.81(6837.10,11765.52) | 9236.72(6964.54,11879.17) | 0.04(0.02,0.06) | 45521.85(40561.25,50555.40) | 49571.75(44592.00,54812.95) | 0.28(0.27,0.30) | 4040.08(2769.97,5667.10) | 4331.37(2968.62,6133.71) | 0.22(0.20,0.24) |
| Namibia | 11036.80(8385.50,14082.26) | 11048.52(8305.10,14222.64) | 0.04(0.03,0.05) | 50627.28(45530.46,56174.37) | 54042.59(48812.32,59546.48) | 0.21(0.20,0.22) | 4739.34(3257.35,6738.33) | 4999.23(3473.27,7088.47) | 0.20(0.19,0.21) |
| Nauru | 11132.44(8381.24,14267.33) | 11315.91(8572.76,14520.43) | 0.06(0.04,0.07) | 55028.37(49901.59,60426.13) | 58026.23(52738.54,63447.39) | 0.17(0.15,0.18) | 4935.40(3344.66,7009.36) | 5092.70(3447.54,7255.68) | 0.11(0.09,0.12) |
| Nepal | 13187.90(9825.59,16994.47) | 12904.64(9631.43,16749.00) | -0.06(-0.08,-0.03) | 60967.02(54767.86,67961.23) | 62543.28(56424.09,69234.48) | 0.11(0.09,0.13) | 6535.60(4696.01,9035.28) | 6633.66(4725.29,9152.20) | 0.10(0.06,0.14) |
| Netherlands | 12019.85(9189.51,15344.47) | 12421.34(9448.85,15880.66) | 0.15(0.04,0.25) | 58712.86(54101.42,63885.41) | 59003.84(53986.14,64260.42) | -0.01(-0.06,0.03) | 5596.69(3917.53,7935.47) | 5494.94(3783.32,7829.15) | -0.05(-0.10,-0.01) |
| New Zealand | 15616.86(11941.50,20005.33) | 15654.22(11910.14,19997.04) | 0.02(-0.01,0.06) | 63855.14(57970.15,70113.98) | 65609.41(59847.24,71435.11) | 0.10(0.09,0.12) | 6412.47(4483.61,9029.41) | 6543.58(4528.13,9373.02) | 0.07(0.04,0.11) |
| Nicaragua | 10475.72(7911.14,13450.48) | 10511.12(7931.06,13542.86) | 0.03(0.02,0.03) | 55169.04(49843.14,60750.33) | 59184.47(54107.93,64567.35) | 0.23(0.22,0.24) | 5227.87(3617.98,7343.38) | 5608.47(3918.03,7820.97) | 0.25(0.24,0.27) |
| Niger | 10752.60(8153.83,13890.35) | 10839.61(8127.98,14052.26) | 0.04(0.02,0.07) | 48461.90(43234.45,54128.19) | 49730.84(44329.07,55391.04) | 0.09(0.09,0.10) | 4407.60(3037.69,6251.24) | 4531.23(3111.17,6424.64) | 0.12(0.10,0.13) |
| Nigeria | 11858.12(8969.70,15209.54) | 11577.92(8804.57,14842.48) | -0.08(-0.11,-0.04) | 53351.54(48019.60,58974.37) | 55396.98(50284.29,60919.60) | 0.14(0.12,0.17) | 4903.49(3377.86,6959.66) | 5055.05(3484.89,7191.71) | 0.13(0.11,0.16) |
| Niue | 11014.06(8314.83,14138.04) | 11160.25(8400.97,14380.46) | 0.07(0.05,0.08) | 55548.32(50383.75,60942.55) | 59019.86(53904.30,64229.86) | 0.20(0.19,0.21) | 4968.38(3375.92,7064.85) | 5164.98(3533.35,7350.77) | 0.14(0.13,0.15) |
| North Macedonia | 13590.05(10125.71,17587.60) | 13696.74(10235.65,17746.60) | 0.02(0.01,0.04) | 56440.37(50439.67,63030.50) | 59214.59(53285.80,65581.00) | 0.18(0.17,0.18) | 5399.63(3675.92,7697.89) | 5597.12(3810.92,7983.54) | 0.15(0.14,0.16) |
| Northern Mariana Islands | 11237.52(8478.53,14439.57) | 11204.98(8478.63,14323.50) | -0.01(-0.02,0.00) | 56812.61(51766.27,62227.43) | 58612.12(53541.48,63803.50) | 0.08(0.06,0.09) | 5126.17(3523.35,7345.43) | 5188.61(3524.14,7486.43) | 0.01(0.00,0.03) |
| Norway | 13221.70(10101.43,16865.91) | 13180.57(10074.05,16796.73) | -0.08(-0.11,-0.04) | 59563.32(54328.93,65051.33) | 60159.01(55072.88,65390.92) | 0.04(0.01,0.08) | 5838.06(4084.20,8242.27) | 5676.10(3910.99,8114.71) | -0.13(-0.15,-0.10) |
| Oman | 11473.02(8569.90,14822.62) | 11647.34(8774.91,15047.12) | 0.05(0.05,0.06) | 53811.82(48205.62,59698.47) | 59316.11(53927.43,64932.81) | 0.33(0.32,0.34) | 5026.22(3441.13,7119.99) | 5572.47(3845.66,7852.58) | 0.38(0.36,0.39) |
| Pakistan | 12283.71(9042.24,16051.61) | 12818.57(9460.03,16704.11) | 0.18(0.16,0.20) | 57199.67(51478.56,63460.27) | 62357.79(56724.49,68640.46) | 0.32(0.29,0.34) | 5871.17(4157.13,8088.04) | 6486.22(4635.25,8953.33) | 0.37(0.34,0.40) |
| Palau | 11004.85(8261.70,14152.26) | 11202.77(8482.56,14299.64) | 0.06(0.05,0.07) | 55335.13(50211.01,60557.27) | 58618.36(53452.76,63951.94) | 0.17(0.16,0.19) | 4867.35(3300.60,6974.70) | 5040.40(3422.61,7294.27) | 0.11(0.09,0.12) |
| Palestine | 11571.48(8710.76,14912.14) | 11643.16(8814.57,15073.54) | 0.01(0.00,0.02) | 53331.12(47693.12,59408.56) | 56969.54(51330.35,62956.46) | 0.17(0.15,0.20) | 5038.65(3411.24,7191.95) | 5279.86(3603.09,7504.48) | 0.10(0.06,0.13) |
| Panama | 10314.46(7741.23,13255.36) | 10451.00(7917.50,13461.22) | 0.04(0.03,0.05) | 57375.31(52019.41,62778.54) | 61515.74(56406.89,67073.77) | 0.22(0.21,0.22) | 5486.47(3840.44,7688.06) | 5900.04(4160.17,8292.02) | 0.23(0.22,0.24) |
| Papua New Guinea | 10734.39(8086.30,13868.30) | 10866.40(8233.78,13936.58) | 0.06(0.04,0.08) | 49488.60(44330.52,55148.50) | 51460.49(46290.12,56993.23) | 0.13(0.13,0.14) | 4384.38(2977.16,6258.52) | 4465.73(3027.19,6469.85) | 0.08(0.07,0.09) |
| Paraguay | 10229.22(7760.80,13035.76) | 10731.01(8104.92,13821.85) | 0.14(0.12,0.16) | 57436.29(52429.81,62468.43) | 60286.31(55262.24,65771.60) | 0.14(0.13,0.16) | 5236.32(3615.97,7386.34) | 5705.00(3970.76,8083.37) | 0.27(0.26,0.29) |
| Peru | 9130.37(6963.84,11595.68) | 9483.12(7244.59,12010.69) | 0.16(0.14,0.17) | 55875.09(51075.39,60852.99) | 59496.53(54598.04,64480.17) | 0.21(0.20,0.22) | 4865.84(3336.80,6982.72) | 5278.98(3658.99,7489.36) | 0.28(0.27,0.29) |
| Philippines | 10960.37(8273.52,14092.33) | 11089.60(8421.10,14208.00) | 0.00(-0.02,0.02) | 50085.73(44908.93,55770.04) | 52914.38(47741.51,58355.76) | 0.14(0.12,0.16) | 4688.18(3236.90,6605.67) | 4888.61(3378.23,6924.52) | 0.09(0.07,0.12) |
| Poland | 15386.36(11652.13,19761.70) | 15279.80(11599.63,19472.48) | -0.05(-0.06,-0.04) | 61207.49(54943.30,67872.57) | 63114.33(57175.09,69215.40) | 0.10(0.09,0.11) | 6300.92(4394.92,8833.18) | 6257.25(4305.71,8885.67) | -0.04(-0.07,-0.00) |
| Portugal | 13395.02(10065.87,17250.75) | 13813.61(10544.40,17585.66) | 0.05(0.02,0.08) | 58614.31(53093.74,64414.82) | 61790.39(56531.25,67302.43) | 0.23(0.16,0.30) | 5642.45(3865.82,8055.86) | 6038.58(4178.31,8593.48) | 0.23(0.17,0.29) |
| Puerto Rico | 9590.31(7289.28,12246.75) | 9785.73(7487.81,12440.22) | 0.06(0.05,0.07) | 55421.21(50589.16,60553.68) | 58243.14(53356.65,63258.86) | 0.17(0.16,0.17) | 4711.63(3182.98,6849.12) | 4972.56(3398.74,7191.91) | 0.17(0.15,0.19) |
| Qatar | 11692.81(8904.26,15126.02) | 11995.61(9080.48,15476.97) | 0.10(0.09,0.11) | 58131.18(52830.72,63836.66) | 61857.06(56637.78,67415.30) | 0.20(0.19,0.21) | 5435.89(3718.74,7704.76) | 5786.07(4002.05,8096.81) | 0.22(0.22,0.23) |
| Republic of Korea | 12692.96(9725.48,16231.78) | 12448.19(9550.21,15874.62) | -0.04(-0.07,0.00) | 66219.70(60976.56,71718.14) | 69152.60(64067.16,74344.69) | 0.18(0.12,0.24) | 6166.23(4200.96,8961.60) | 6444.63(4439.24,9331.37) | 0.17(0.10,0.23) |
| Republic of Moldova | 13770.36(10415.82,17724.33) | 13857.72(10403.92,17799.89) | 0.02(0.01,0.03) | 56538.14(50212.94,63322.70) | 60608.72(54626.06,66800.09) | 0.25(0.23,0.27) | 5399.86(3650.69,7772.07) | 5885.03(4048.54,8358.61) | 0.28(0.25,0.30) |
| Romania | 14249.24(10684.01,18263.83) | 14136.05(10541.29,18209.44) | -0.03(-0.04,-0.02) | 58422.19(51905.41,65456.07) | 60054.08(53798.17,66683.44) | 0.10(0.10,0.11) | 5764.36(3917.75,8203.61) | 5901.03(4059.22,8458.71) | 0.08(0.08,0.09) |
| Russian Federation | 15229.34(11507.81,19473.73) | 15340.12(11661.63,19576.44) | 0.04(0.03,0.04) | 62978.60(56874.31,69305.29) | 64356.43(58465.66,70482.40) | 0.15(0.12,0.18) | 6116.83(4187.47,8719.73) | 6341.80(4383.73,8992.95) | 0.20(0.14,0.25) |
| Rwanda | 12063.16(9012.03,15655.70) | 11850.22(8858.61,15214.60) | -0.06(-0.08,-0.05) | 51072.69(45477.06,57417.01) | 50418.63(44623.95,56882.29) | 0.04(0.00,0.08) | 4884.49(3351.82,6947.01) | 4662.22(3144.39,6725.58) | -0.02(-0.07,0.04) |
| Saint Kitts and Nevis | 9588.16(7207.01,12316.66) | 9700.35(7347.63,12370.68) | 0.03(0.03,0.04) | 53995.21(49222.17,58936.92) | 57131.26(52218.23,61986.80) | 0.18(0.17,0.19) | 4789.69(3343.93,6803.85) | 5151.70(3622.96,7311.77) | 0.24(0.21,0.27) |
| Saint Lucia | 9725.12(7367.75,12478.73) | 9780.44(7414.77,12439.50) | 0.01(0.00,0.02) | 52306.28(47382.82,57460.04) | 56112.57(51283.63,61055.15) | 0.21(0.20,0.23) | 4592.11(3183.94,6506.14) | 4897.80(3382.54,6961.06) | 0.17(0.15,0.18) |
| Saint Vincent and the Grenadines | 9452.23(7179.70,12192.34) | 9634.83(7339.98,12313.94) | 0.07(0.06,0.07) | 51776.23(46837.47,56845.50) | 55413.31(50463.72,60413.84) | 0.23(0.22,0.24) | 4445.64(3054.80,6413.03) | 4815.18(3346.02,6926.74) | 0.25(0.24,0.27) |
| Samoa | 11362.80(8527.62,14613.35) | 11297.97(8542.84,14537.25) | -0.03(-0.04,-0.02) | 55561.84(50433.27,61117.40) | 57748.36(52470.48,63132.48) | 0.11(0.10,0.12) | 5058.94(3443.74,7161.45) | 5079.56(3470.30,7290.88) | -0.02(-0.04,-0.00) |
| San Marino | 13043.18(9858.68,16763.51) | 13038.83(9855.38,16738.14) | -0.01(-0.03,0.01) | 59799.67(54357.69,65250.65) | 60685.84(55452.06,66239.29) | 0.04(0.03,0.05) | 5679.52(3877.89,8126.43) | 5664.20(3881.07,8208.93) | -0.01(-0.03,0.00) |
| Sao Tome and Principe | 10298.84(7758.15,13255.62) | 10341.89(7857.93,13275.44) | 0.01(-0.00,0.03) | 50645.00(45649.37,55937.89) | 54740.81(49888.56,59976.56) | 0.27(0.26,0.28) | 4516.59(3080.22,6421.14) | 4767.26(3283.10,6838.26) | 0.20(0.19,0.21) |
| Saudi Arabia | 11452.92(8615.03,14868.81) | 11844.28(8994.10,15264.66) | 0.12(0.11,0.13) | 53562.66(47764.43,59519.81) | 59125.42(53707.30,64831.83) | 0.24(0.21,0.28) | 4884.34(3318.55,6935.24) | 5475.47(3742.11,7747.97) | 0.35(0.33,0.37) |
| Senegal | 10603.05(8020.22,13643.32) | 10358.45(7814.38,13351.24) | -0.09(-0.11,-0.07) | 50124.06(45039.16,55531.06) | 51998.01(46811.55,57198.76) | 0.09(0.08,0.10) | 4494.10(3097.11,6338.23) | 4589.84(3147.56,6495.11) | 0.04(0.03,0.05) |
| Serbia | 14014.14(10475.24,18084.70) | 14163.05(10563.55,18338.75) | 0.03(0.02,0.03) | 57814.50(51645.25,64730.13) | 60968.19(54732.64,67501.54) | 0.19(0.18,0.20) | 5759.25(3957.73,8173.32) | 6049.28(4122.82,8613.67) | 0.17(0.16,0.18) |
| Seychelles | 9870.71(7426.80,12842.86) | 9833.69(7419.85,12640.79) | -0.00(-0.01,0.00) | 51684.45(46576.07,56942.60) | 55232.92(50179.78,60398.35) | 0.20(0.19,0.21) | 4678.95(3199.14,6622.87) | 4843.32(3303.12,6836.95) | 0.10(0.09,0.11) |
| Sierra Leone | 10973.66(8214.36,14241.29) | 10738.88(8047.52,13810.42) | -0.07(-0.09,-0.06) | 50100.68(44877.52,55776.53) | 51918.78(46599.19,57466.55) | 0.13(0.11,0.15) | 4601.18(3170.95,6527.31) | 4665.02(3209.91,6639.87) | 0.07(0.05,0.10) |
| Singapore | 11676.82(8910.04,14972.77) | 11349.83(8746.29,14464.21) | -0.03(-0.07,0.00) | 66439.51(61385.89,71611.18) | 68142.72(63198.19,73131.42) | 0.10(0.09,0.11) | 6159.43(4241.68,8918.48) | 6256.81(4268.86,9097.59) | 0.09(0.07,0.12) |
| Slovakia | 14466.86(10952.20,18624.63) | 14250.86(10686.81,18285.26) | -0.05(-0.06,-0.03) | 60955.07(54521.53,67783.53) | 62053.75(55978.15,68442.64) | 0.07(0.05,0.10) | 5969.58(4101.33,8454.17) | 6001.61(4099.67,8652.21) | 0.05(0.01,0.09) |
| Slovenia | 13733.53(10292.25,17621.46) | 13822.03(10321.87,17875.04) | -0.00(-0.01,0.01) | 57835.53(51759.03,64342.97) | 60464.79(54479.75,66780.06) | 0.13(0.12,0.15) | 5566.71(3819.64,7952.87) | 5832.53(3999.18,8341.02) | 0.13(0.11,0.14) |
| Solomon Islands | 10818.88(8165.43,13936.40) | 11254.95(8515.26,14479.66) | 0.17(0.15,0.19) | 49881.95(44676.60,55605.07) | 53469.26(48113.80,59156.16) | 0.24(0.23,0.25) | 4342.15(2930.62,6271.69) | 4590.17(3132.83,6645.57) | 0.22(0.20,0.23) |
| Somalia | 11667.35(8669.68,15164.99) | 11592.08(8627.38,15055.58) | -0.01(-0.03,0.00) | 49713.11(44172.04,55746.46) | 50071.58(44600.97,56024.88) | 0.03(0.03,0.03) | 4596.41(3141.98,6537.45) | 4614.86(3153.60,6586.07) | 0.02(0.02,0.03) |
| South Africa | 12056.83(9148.02,15458.28) | 11380.16(8681.40,14578.79) | -0.14(-0.15,-0.12) | 56303.90(51014.46,61813.67) | 57584.25(52499.03,62816.46) | 0.11(0.10,0.13) | 5272.86(3658.07,7495.21) | 5223.86(3640.20,7370.45) | 0.03(0.01,0.05) |
| South Sudan | 11659.51(8659.68,15114.73) | 11495.83(8583.04,14861.07) | -0.06(-0.08,-0.04) | 50367.23(44849.08,56558.12) | 50460.28(45038.01,56568.77) | 0.01(0.01,0.02) | 4580.24(3154.98,6477.61) | 4549.10(3149.79,6458.67) | -0.01(-0.02,-0.00) |
| Spain | 12527.53(9868.77,15591.80) | 12567.47(9415.28,16342.27) | 0.09(-0.00,0.19) | 58300.79(53682.26,63036.22) | 58518.13(53375.66,63757.72) | -0.03(-0.09,0.02) | 5691.31(4057.08,7930.93) | 5257.18(3588.68,7633.36) | -0.30(-0.47,-0.13) |
| Sri Lanka | 9890.47(7439.18,12688.37) | 9835.54(7376.46,12717.97) | -0.01(-0.02,0.01) | 49117.39(44057.27,54529.84) | 51747.22(46646.39,57100.12) | 0.17(0.16,0.19) | 4424.11(3043.66,6171.99) | 4521.95(3080.25,6426.96) | 0.08(0.06,0.10) |
| Sudan | 11380.38(8503.79,14814.16) | 11638.66(8799.25,14964.92) | 0.07(0.07,0.08) | 51242.21(45657.87,57261.87) | 56479.51(51074.80,62186.88) | 0.33(0.32,0.35) | 4931.35(3377.42,6929.88) | 5441.12(3742.06,7708.43) | 0.34(0.33,0.36) |
| Suriname | 9548.43(7249.01,12263.65) | 9768.07(7419.11,12564.48) | 0.08(0.06,0.09) | 53632.55(48760.76,58723.60) | 56335.67(51433.44,61347.05) | 0.17(0.17,0.18) | 4605.45(3148.03,6657.07) | 4799.48(3299.72,6876.87) | 0.16(0.15,0.17) |
| Sweden | 10237.18(7987.87,12781.98) | 11759.57(8779.60,15281.06) | 0.51(0.39,0.63) | 51676.50(46623.42,56619.67) | 56191.39(50650.89,61830.16) | 0.33(0.20,0.46) | 4735.68(3283.04,6738.09) | 5289.65(3617.33,7550.83) | 0.38(0.25,0.51) |
| Switzerland | 12985.49(10044.20,16361.70) | 13456.63(10305.67,17260.92) | 0.19(0.15,0.22) | 57782.43(52762.84,63149.30) | 60383.66(54864.40,66111.09) | 0.19(0.17,0.20) | 5484.76(3826.91,7725.64) | 5806.93(3981.78,8303.53) | 0.28(0.24,0.32) |
| Syrian Arab Republic | 11589.14(8718.86,14898.97) | 11725.21(8822.57,15122.10) | 0.02(0.01,0.03) | 54048.47(48468.18,59840.79) | 56244.00(50495.15,62191.93) | 0.14(0.10,0.18) | 5150.36(3523.07,7240.73) | 5180.80(3507.33,7379.77) | 0.04(-0.03,0.10) |
| Taiwan (Province of China) | 11419.46(9203.81,13753.68) | 12380.24(10083.30,14727.04) | 0.37(0.30,0.44) | 57131.01(52765.43,61559.36) | 63265.12(59892.89,66808.73) | 0.39(0.35,0.43) | 5304.60(3729.14,7401.99) | 6205.06(4468.35,8554.68) | 0.64(0.57,0.71) |
| Tajikistan | 12399.99(9212.28,16146.13) | 12358.84(9195.48,15931.58) | -0.03(-0.03,-0.02) | 54160.99(48251.99,60419.42) | 57098.74(51253.98,63077.52) | 0.21(0.18,0.25) | 5018.28(3387.04,7244.55) | 5193.48(3524.20,7474.68) | 0.17(0.14,0.20) |
| Thailand | 9172.70(6917.26,11858.57) | 9516.27(7177.81,12274.63) | 0.16(0.12,0.20) | 50469.61(45400.22,55693.68) | 54816.42(49703.18,60172.18) | 0.28(0.27,0.28) | 4706.48(3280.97,6501.33) | 5074.83(3537.09,7152.22) | 0.25(0.24,0.26) |
| Timor-Leste | 9760.20(7312.09,12666.75) | 9590.16(7207.49,12342.19) | -0.05(-0.06,-0.03) | 46188.86(41146.30,51767.50) | 49710.15(44645.45,55155.28) | 0.28(0.26,0.30) | 4178.06(2860.86,5865.36) | 4445.41(3036.93,6288.53) | 0.26(0.22,0.30) |
| Togo | 10991.91(8224.04,14294.01) | 10750.16(8115.40,13897.55) | -0.10(-0.13,-0.07) | 50486.47(44934.10,56458.52) | 52541.88(47443.09,57820.02) | 0.11(0.11,0.12) | 4629.80(3163.51,6583.92) | 4754.71(3308.35,6700.51) | 0.07(0.05,0.08) |
| Tokelau | 10936.28(8214.41,14142.15) | 11156.44(8454.71,14442.46) | 0.08(0.06,0.09) | 53461.01(48407.08,58836.25) | 57795.71(52794.09,63147.40) | 0.26(0.25,0.27) | 4785.03(3232.29,6783.81) | 5053.65(3440.20,7239.22) | 0.20(0.19,0.21) |
| Tonga | 11191.69(8383.45,14493.19) | 11245.57(8536.71,14440.31) | 0.00(-0.01,0.02) | 54640.63(49454.76,60176.57) | 57556.08(52369.34,63045.39) | 0.14(0.12,0.16) | 5033.09(3455.95,7114.61) | 5184.60(3569.42,7372.74) | 0.07(0.05,0.09) |
| Trinidad and Tobago | 9680.28(7362.67,12391.00) | 9829.47(7455.52,12519.17) | 0.05(0.04,0.06) | 54494.61(49688.35,59652.88) | 57502.11(52468.65,62613.29) | 0.19(0.18,0.20) | 4834.05(3372.92,6836.50) | 5085.51(3552.97,7260.80) | 0.18(0.17,0.19) |
| Tunisia | 11801.79(8920.45,15152.49) | 11968.00(9067.98,15347.31) | 0.06(0.05,0.07) | 55226.26(49824.61,60993.57) | 59407.19(54070.04,65145.74) | 0.24(0.24,0.25) | 5327.65(3629.05,7464.07) | 5701.91(3931.70,8025.63) | 0.25(0.24,0.26) |
| Turkmenistan | 12624.27(9414.92,16409.79) | 12698.98(9546.33,16381.15) | 0.10(0.07,0.13) | 56327.87(50588.85,62554.42) | 61360.75(55216.42,67369.62) | 0.30(0.26,0.33) | 5199.70(3523.81,7542.39) | 5569.25(3759.74,8076.95) | 0.26(0.21,0.31) |
| Tuvalu | 10924.31(8228.16,14120.48) | 11174.59(8456.51,14353.81) | 0.02(0.01,0.03) | 53155.66(48032.76,58614.52) | 57241.47(52148.29,62531.03) | 0.31(0.28,0.34) | 4778.64(3266.40,6771.96) | 5040.77(3444.88,7216.66) | 0.26(0.23,0.29) |
| Turkey | 11370.37(8710.04,14571.30) | 11686.84(8845.13,15046.38) | 0.08(0.07,0.09) | 54131.81(48915.17,59891.65) | 59165.99(53887.08,64697.48) | 0.24(0.23,0.26) | 5274.53(3657.76,7305.72) | 5700.93(3916.47,7956.60) | 0.22(0.20,0.23) |
| Uganda | 11877.43(8871.20,15566.14) | 11641.59(8713.75,15081.30) | -0.06(-0.08,-0.05) | 50262.67(44697.87,56624.77) | 51901.08(46423.83,58004.69) | 0.10(0.10,0.11) | 4669.47(3192.62,6646.96) | 4821.01(3306.61,6852.80) | 0.11(0.10,0.12) |
| Ukraine | 16012.54(12078.77,20497.86) | 15859.65(11962.35,20318.83) | -0.01(-0.02,0.01) | 63144.94(56443.70,70129.75) | 64987.20(58763.83,71547.61) | 0.13(0.11,0.15) | 6367.24(4349.48,9117.49) | 6529.78(4467.92,9293.40) | 0.16(0.12,0.20) |
| United Arab Emirates | 11163.02(8508.48,14365.13) | 11631.80(8834.09,14975.99) | 0.12(0.11,0.14) | 55263.02(49917.38,60912.86) | 60403.24(55224.22,65845.20) | 0.29(0.29,0.30) | 5173.07(3561.03,7275.74) | 5906.28(4170.74,8160.21) | 0.53(0.49,0.57) |
| United Kingdom | 12177.51(9388.32,15363.66) | 13980.81(10715.46,17789.82) | 0.67(0.57,0.77) | 59312.80(54329.55,64515.15) | 63403.93(58302.56,68628.84) | 0.21(0.16,0.27) | 5758.10(4024.39,8119.25) | 6265.35(4341.29,8857.33) | 0.35(0.29,0.41) |
| United Republic of Tanzania | 11825.53(8812.94,15374.66) | 11685.69(8739.94,15139.55) | -0.05(-0.06,-0.03) | 52184.72(46696.79,58377.10) | 52911.01(47628.63,58716.95) | -0.00(-0.02,0.02) | 4815.12(3329.50,6840.39) | 4858.01(3331.74,6931.63) | 0.02(0.01,0.03) |
| United States of America | 12887.16(9963.51,16289.16) | 13092.85(10647.66,15727.04) | 0.08(0.05,0.10) | 65695.66(60769.36,70766.48) | 69639.16(65811.13,73476.73) | 0.12(0.09,0.16) | 6491.47(4571.92,9122.20) | 6879.46(4957.88,9486.55) | 0.13(0.10,0.15) |
| United States Virgin Islands | 9635.52(7303.39,12327.63) | 9789.06(7464.83,12573.52) | 0.05(0.04,0.07) | 55234.55(50441.39,60173.13) | 58132.01(53372.42,63174.13) | 0.16(0.16,0.17) | 4750.73(3234.62,6851.15) | 4946.80(3394.93,7087.29) | 0.13(0.12,0.15) |
| Uruguay | 12696.50(9636.69,16187.83) | 13662.35(10334.81,17585.57) | 0.19(0.14,0.24) | 64529.44(59264.45,70198.51) | 68534.34(63269.22,74100.63) | 0.18(0.15,0.20) | 6521.19(4573.91,9220.69) | 7172.10(4997.81,10038.78) | 0.27(0.23,0.32) |
| Uzbekistan | 12696.33(9503.77,16488.64) | 12848.75(9698.74,16523.64) | 0.04(0.03,0.05) | 57134.44(51334.11,63470.89) | 61511.45(55333.39,67648.31) | 0.25(0.22,0.27) | 5298.35(3573.93,7637.85) | 5667.00(3884.44,8149.01) | 0.26(0.23,0.30) |
| Vanuatu | 11209.03(8421.67,14347.26) | 11397.10(8573.54,14721.58) | 0.06(0.05,0.08) | 52246.96(46952.72,58058.44) | 54959.25(49713.05,60657.52) | 0.16(0.16,0.17) | 4832.66(3304.22,6886.86) | 5015.68(3450.08,7147.44) | 0.13(0.11,0.14) |
| Venezuela (Bolivarian Republic of) | 10245.14(7781.82,13188.57) | 10056.57(7613.26,12827.32) | -0.07(-0.08,-0.06) | 58104.89(52884.56,63514.80) | 60429.62(55325.43,65737.83) | 0.09(0.07,0.11) | 5553.77(3891.15,7809.53) | 5746.87(4047.42,8041.85) | 0.09(0.07,0.10) |
| Viet Nam | 10064.33(7517.01,12966.86) | 9992.75(7531.67,12864.04) | 0.04(0.01,0.07) | 46365.53(41179.23,51813.20) | 49709.27(44469.22,55265.43) | 0.25(0.24,0.27) | 4281.78(2938.45,6000.75) | 4545.68(3116.53,6423.38) | 0.24(0.21,0.27) |
| Yemen | 11485.39(8586.91,14950.02) | 11503.15(8672.18,14836.15) | 0.00(-0.01,0.01) | 50627.73(44805.65,56832.71) | 53865.09(48396.90,59842.63) | 0.25(0.23,0.28) | 4851.25(3313.68,6825.12) | 5081.74(3477.67,7132.76) | 0.20(0.17,0.23) |
| Zambia | 10781.91(8178.81,13741.88) | 11260.20(8531.44,14670.11) | 0.06(0.02,0.09) | 50049.90(45055.22,55340.15) | 52176.13(47009.62,57810.00) | 0.11(0.09,0.13) | 4413.19(3045.30,6279.71) | 4685.27(3210.92,6684.97) | 0.15(0.12,0.17) |
| Zimbabwe | 10951.10(8249.86,14103.56) | 11218.76(8435.82,14421.47) | 0.09(0.08,0.09) | 50388.79(45350.54,55990.65) | 51966.68(46575.30,57745.78) | 0.07(0.05,0.09) | 4682.44(3210.80,6580.35) | 4847.08(3367.26,6828.82) | 0.12(0.10,0.14) |
| CI =confidence interval, DALYs = disability-adjusted life years, GBD = Global Burden of Disease Study, SDI = sociodemographic index,EAPC = Estimated Annual Percentage Change. | | | | | | | | | |
